# Structural features and seismotectonic implications of coseismic surface ruptures produced by the 2016 *M*_w_ 7.1 Kumamoto earthquake

**DOI:** 10.1007/s10950-017-9653-5

**Published:** 2017-03-21

**Authors:** Aiming Lin

**Affiliations:** Department of Geophysics, Graduate School of Science, Kyoto University, Kyoto, 606-8502 Japan

**Keywords:** 2016 *M*_w_ 7.1 Kumamoto earthquake, Coseismic surface rupture, Hinagu–Futagawa fault zone, Aso caldera, Strike-slip fault, Coseismic graben structure

## Abstract

Field investigations and analyses of satellite images and aerial photographs reveal that the 2016 *M*_w_ 7.1 (Mj 7.3) Kumamoto earthquake produced a ∼40-km surface rupture zone striking NE-SW on central Kyushu Island, Japan. Coseismic surface ruptures were characterized by shear faults, extensional cracks, and mole tracks, which mostly occurred along the pre-existing NE-SW-striking Hinagu–Futagawa fault zone in the southwest and central segments, and newly identified faults in the northeast segment. This study shows that (i) the Hinagu–Futagawa fault zone triggered the 2016 Kumamoto earthquake and controlled the spatial distribution of coseismic surface ruptures; (ii) the southwest and central segments were dominated by right-lateral strike-slip movement with a maximum in-site measured displacement of up to 2.5 m, accompanied by a minor vertical component. In contrast, the northeast segment was dominated by normal faulting with a maximum vertical offset of up to 1.75 m with a minor horizontal component that formed graben structures inside Aso caldera; (iii) coseismic rupturing initiated at the jog area between the Hinagu and Futagawa faults, then propagated northeastward into Aso caldera, where it terminated. The 2016 *M*_w_ 7.1 Kumamoto earthquake therefore offers a rare opportunity to study the relationships between coseismic rupture processes and pre-existing active faults, as well as the seismotectonics of Aso volcano.

**Electronic supplementary material:**

The online version of this article (doi:10.1007/s10950-017-9653-5) contains supplementary material, which is available to authorized users.

## Introduction

**Fig. 1:**
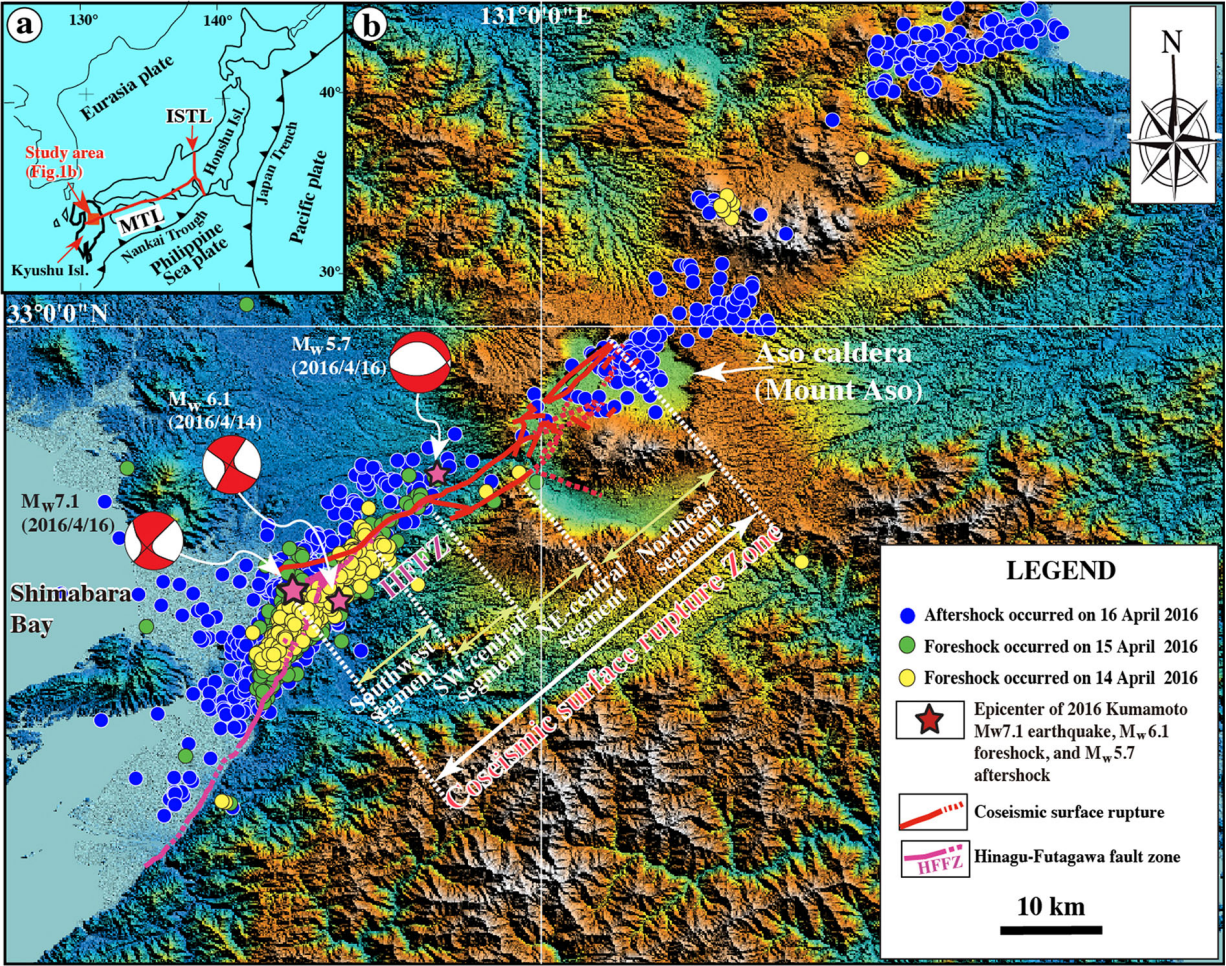
Index map of the study area showing **a** the tectonic setting and **b** color-shaded relief map showing the distribution of the coseismic surface ruptures, foreshocks, and aftershocks that occurred in the period during 14 and 16 April 2016 [modified from Lin et al. (2016)]. Active fault data are from RGAFJ (1980, 1991) and Geographical Survey Institute (2001). Epicenter data and focal mechanisms are from the Disaster Information Laboratory (2016). *MTL* Median Tectonic Line, *ISTL* Itoigawa–Shizuoka Tectonic Line, *Honshu Isl.* Honshu Island, *Kyushu Isl.* Kyushu Island, *HFFZ* Hinagu–Futagawa fault zone

The *M*_w_ 7.1 (Mj 7.3) Kumamoto earthquake occurred on 16 April 2016 (Fig. 1), resulting in extensive damage and more than 50 deaths on Kyushu Island, Japan. With a seismic rupture zone ≥40 km long and a magnitude (Mj) of 7.3, this shock was the largest inland earthquake recorded in Japan Islands in the past century. The main shock was accompanied by >1000 foreshocks and aftershocks during the week of 14–20 April 2016 (Japan Meteorological Agency 2016a). Three *M*_w_ ≥5.5 foreshocks occurred 2 days before the main shock, including an *M*_w_ 6.2 (Mj 6.5) and *M*_w_ 5.5 (Mj 5.7) on 14 April 2016 and an *M*_w_ 6.0 (Mj 6.4) on 15 April 2016. Subsequently, four *M*_w_ >5.0 aftershocks occurred within 6 h of the main shock on 16 April 2016 (Japan Meteorological Agency 2016a). Epicenters migrated from southwest to northeast, mostly along pre-existing active faults and throughout Aso caldera (Fig. 1b). A maximum seismic intensity of 7 (on the Japanese seven-point seismic intensity scale) was observed at both the epicenters of the largest foreshock (*M*_w_ 6.2) and the *M*_w_ 7.1 main shock (Japan Meteorological Agency 2016a), indicating severe damage throughout central Kyushu Island, including structures in the Aso caldera region. A previous study reveals that the coseismic surface rupturing terminated at Aso caldera, and suggests that the newly formed coseismic ruptures under Aso caldera are potential new channels for magma venting, which change the spatial heterogeneity and mechanical property of Aso volcano, therefore may require reassessing the volcanic hazard in the vicinity of Aso volcano (Lin et al. 2016). Amazingly, as suggested in a previous study (Lin et al. 2016), Aso volcano re-erupted on 8 October 2016 after a 36-year dormant duration (Japan Meteorological Agency 2016b).

Seismic inversion results suggest that (i) the focal depth was ∼15 km and (ii) the earthquake had a predominantly strike-slip focal mechanism on a fault striking NE-SW and dipping SE at ∼80°, with a compression axis oriented E-W (National Research Institute for Earth Science and Disaster Prevention 2016, Yagi et al. 2016). The seismic source inversions for the earthquake sequences of *M* >6 foreshocks and *M*_w_ 7.1 main shock show that (i) the total length of fault ruptured zone is up to 40∼50 km and (ii) the southwestern segment of the seismogenic fault was dominated by right-lateral strike-slip mechanism and the northeastern segment had a combination feature of strike-slip and normal faults (Asono and Iwata 2016; Kubo et al. 2016). These seismic results are consistent with the field observations that (i) the total length of coseismic surface rupture zone is up to ∼40 km, (ii) the surface deformation in the southwestern segment of the rupture zone is dominated by strike-slip displacement, and (iii) the northeastern segment of the rupture zone was characterized by normal-dominated displacement that formed graben structures extending ∼10 km within the west-southwest side of Aso caldera (Lin et al. 2016). An *M*_w_ 5.7 aftershock in the Aso caldera area on 16 April 2016 also exhibited normal faulting, consistent with field observations of fault structures (Fig. 1b; Japan Meteorological Agency 2016a; National Research Institute for Earth Science and Disaster Prevention 2016).

In order to determine the motion of the seismogenic fault, ground deformation, and relationships between coseismic surface ruptures and pre-existing faults, our survey group conducted a 10-day field study of structural features, beginning 1 day after the main shock. During this time, we observed the principal structural features and measured offsets at the main locations of the coseismic surface ruptures, and then retrieved ground deformation markers from locations that were damaged during the earthquake. The preliminary field works have been reported in our previous paper (Lin et al. 2016). Subsequent fieldwork in surface rupture zones has been conducted continuously in the mountains and Aso caldera for the last 6 months.

In this study, I focus on structural features, including distribution patterns and coseismic surface rupture offsets. I discuss the relationships between surface ruptures and pre-existing active faults, as well as their seismotectonic implications.

## Tectonic setting

The study area is located in central Kyushu Island, around the west and central side of Aso caldera, southwest Japan (Fig. 1). Mount Aso is one of the largest active volcanoes on the Earth, with a caldera area of ∼380 km^2^. Activity initiated at the Aso volcanic cluster ∼0.3 Myr ago with a large eruption that generated extensive pyroclastic flows. Four subsequent large, explosive eruptions resulted in the formation of Aso caldera (Ono and Watanabe 1985; Okubo and Shibuya 1993). Pyroclastic flows and volcanic ash from the caldera-forming eruption sequence covered a wide region of central Kyushu, including the study area. The basement rocks of the study area are mainly composed of Paleozoic metamorphic rocks, non- or weakly metamorphic eugeosynclinal rocks, Mesozoic granitic rocks, and marine sediments (Ono and Watanabe 1985).

The Hinagu–Futagawa fault zone is the western extension of the Median Tectonic Line (e.g., Kamata and Kodama 1994; Takagi et al. 2007; Matsumoto et al. 2015), which is composed of the NNE-SSW- to NE-SW-striking Hinagu Fault and NE-SW- to ENE-WSW-striking Futagawa Fault, and extends for ∼81 km in the central Kyushu Island (Fig. 1). Previous studies have shown that both the Hinagu and Futagawa faults are currently active, with recurrence intervals for large earthquakes of 3600–11,000 years for the Hinagu Fault and 2600–8100 years for the Futagawa Fault. The most recent event on the Hinagu Fault occurred between 1200 and 1600 years BP, whereas the most recent large event on the Futagawa Fault occurred 2200 years BP (Headquarters for Earthquake Research Promotion 2016). The 2016 Kumamoto earthquake occurred in the jog area between the Hinagu and Futagawa faults, ∼30 km southwest of Aso caldera (Fig. 1b; Japan Meteorological Agency 2016a; Geospatial Information Authority of Japan 2016). Historical and instrumental records show that >10 large earthquakes (*M* ≥ 6.0) have occurred in the central Kyushu Island around the study area since AD 679 (Headquarters for Earthquake Research Promotion 2016). Seismic and geological data show that the Hinagu–Futagawa fault zone is currently active and its earthquakes frequently cause severe damage, as exemplified by the 2016 Kumamoto main shock (Headquarters for Earthquake Research Promotion 2016). Previous studies show that the minimum principal compressive stress (*σ*_3_) is oriented N-S to NNW-SSE in Kyushu Island, indicating a principal maximum compressive stress (*σ*_1_) of E-W to WNW-ESE in the study area around the Hinagu–Futagawa fault zone (Itoh et al. 2014; Matsumoto et al. 2015). This stress direction is considered to be associated with the ongoing penetration of the Philippine Sea Plate into the Eurasian Plate (Matsumoto et al. 2015).

## Structural analyses of coseismic surface ruptures

### Terminology

The term *coseismic surface rupture* is defined as a surface fracture produced by a current or large historic earthquake. The term is interchangeable with *surface earthquake fault* and *earthquake fault* in Japan, which is enhanced for the topographic morphology and geometry of surface fractures formed during large earthquakes (Research Group for Active Faults of Japan (RGAFJ) 1980, 1991). In general, it is difficult to understand whether or not surface ruptures, including slope failures and landslides, are directly caused by seismogenic faulting or strong ground shaking during individual earthquakes. In this study, to avoid any confusion regarding the terminology, we use the term *coseismic surface rupture* for the surface faults, fractures, cracks, and mole tracks that occurred during the 2016 Kumamoto earthquake, apart from when referring to distinct slope failures and landslides that occurred locally.

### Study methods

To detect and identify tectonic-related topographic features in the study area, we examined aerial photographs acquired before and after the 2016 Kumamoto earthquake, color-shaded relief maps generated from 1:25,000 DEM data with a 10-m mesh grid, and high-resolution Google Earth images acquired on 18 April 2016 after the main shock. Aerial photographs and topographical maps were provided by the Geospatial Information Authority of Japan (2016). Given that images were available both before and after the main shock, it is possible to determine which ground deformation features and tectonic-related topographic features in the study area are directly related with coseismic deformation caused by the 2016 Kumamoto earthquake. Tectonic-related topographic features identified using these methods were confirmed in the field. Our fieldwork was guided by topographic maps, aerial photographs, and high-resolution Google Earth images acquired shortly after the main shock.

Coseismic displacements along surface ruptures were measured by a tape measure from offsets of linear surface markers, such as roads, field paths, gullies, and river channels, using the method of Lin and Uda (1996). In mountainous areas, where access was difficult due to road damage from the Kumamoto earthquake, surface ruptures were mostly identified from aerial photographs acquired by the Geospatial Information Authority of Japan (2016) 1 to 2 days after the main shock, and high-resolution Google Earth images acquired on 18 April 2016, 2 days after the main shock. Interferometric Synthetic Aperture Radar (InSAR) data were also used in this study for comparing the deformation features of ground surfaces along the coseismic surface ruptures observed in the field and detected by the observation data acquired in April 2016 before and after the earthquake, that were released by Geospatial Information Authority of Japan (2016).

### Distribution of coseismic surface ruptures

Field investigations reveal that the 2016 *M*_w_ 7.1 Kumamoto earthquake produced a ∼40-km-long surface rupture zone striking NE-SW in the central part of Kyushu Island, from the east side of Shimabara Bay in the southwest to Aso caldera in the northeast (Fig. 1b). Based on structural features and distribution patterns of coseismic surface ruptures, the rupture zone can be divided into four segments, from southwest to northeast, which are the southwest, southwest-central (SW-central), northeast-central (NE-central), and northeast segments (Fig. 1b). The locations of surface ruptures, with detailed longitude and latitude information corresponding to field observations of structural features and analyses of aerial photographs and Google Earth images, are given in Table S1.

**Fig. 2:**
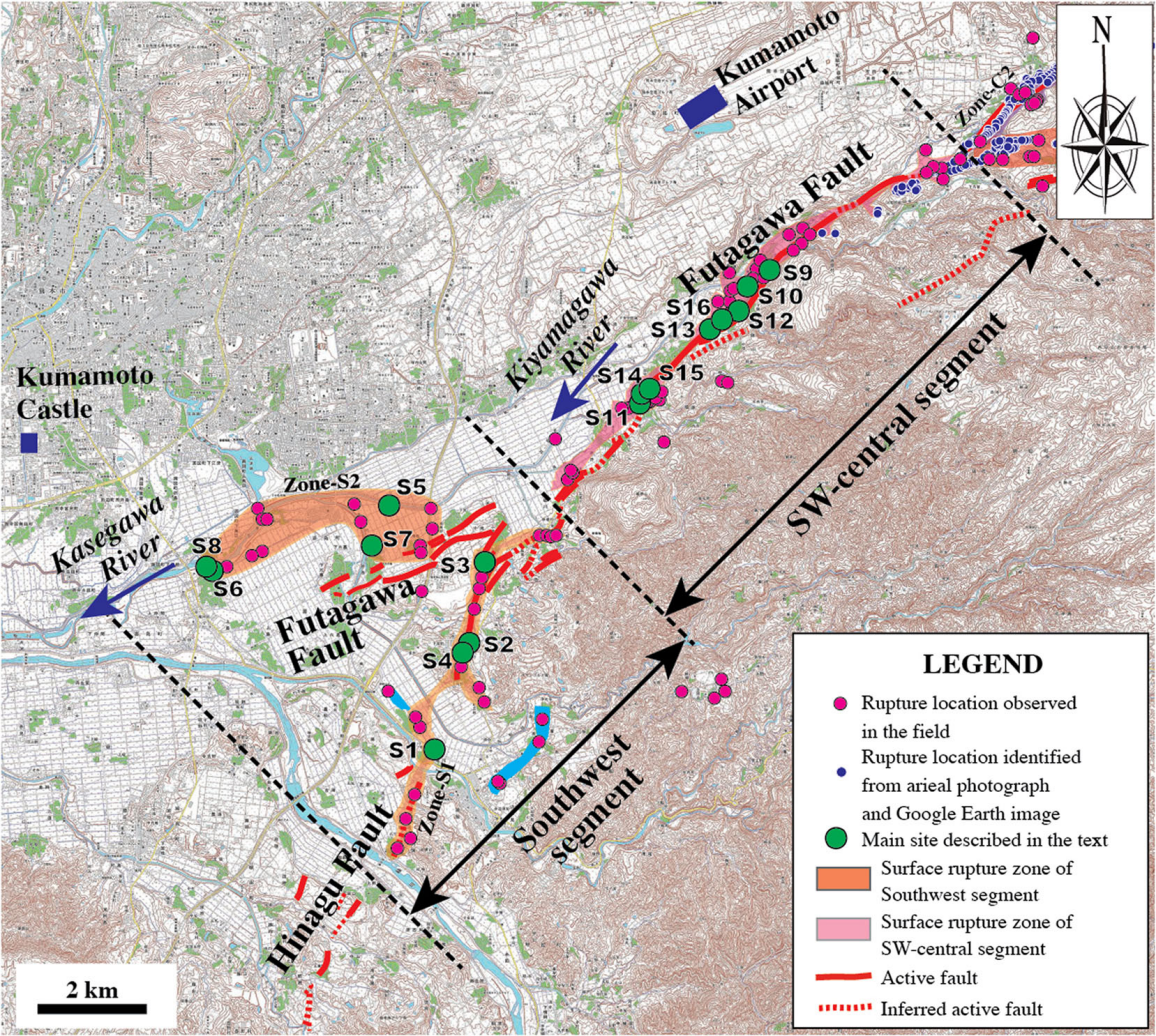
Topographic map showing the distribution of coseismic surface ruptures along the southwest and SW-central segments. Active fault data are from RGAFJ (1980, 1991) and Geographical Survey Institute (2001) (using 1:25,000 topographical map released by the Geographical Survey Institute)

The southwest segment branched into two subrupture zones. One occurred mostly along the main fault trace of the Hinagu Fault (called Zone-S1), along the topographic boundary between lowlands in the west and mountain slopes in the east, striking N10–30° E. The other (called Zone-S2) was distributed across the lowlands bounded by the Kasegawa River, striking N70–80° E, oblique to the trace of the Futagawa Fault at an angle of 10–30° (Fig. 2). The ruptures of Zone-S1 were concentrated in a zone of width <30 m (generally 3–10 m) along the trace of the Hinagu Fault. In contrast, the surface ruptures in Zone-S2 were dispersed across a zone >100 m wide (Fig. 2).

The SW-central segment occurred mostly along the main trace of the Futagawa Fault, striking N50–60° E, which developed along the topographic boundary between the Kiyamagawa River lowlands and the southwestern slope of Mount Aso (Figs. 1 and 2). The ruptures were generally concentrated in a zone ranging from 2–3 to ∼100 m in width (typically 5–10 m). Locally, WSW-ENE- to E-W-striking surface ruptures with distinct shear faults occurred over a wide area that are 50–100 m from the NE-SW-striking rupture zone, which forms a conjugate rupture structure to the NE-SW-striking ruptures (see below for details).

**Fig. 3:**
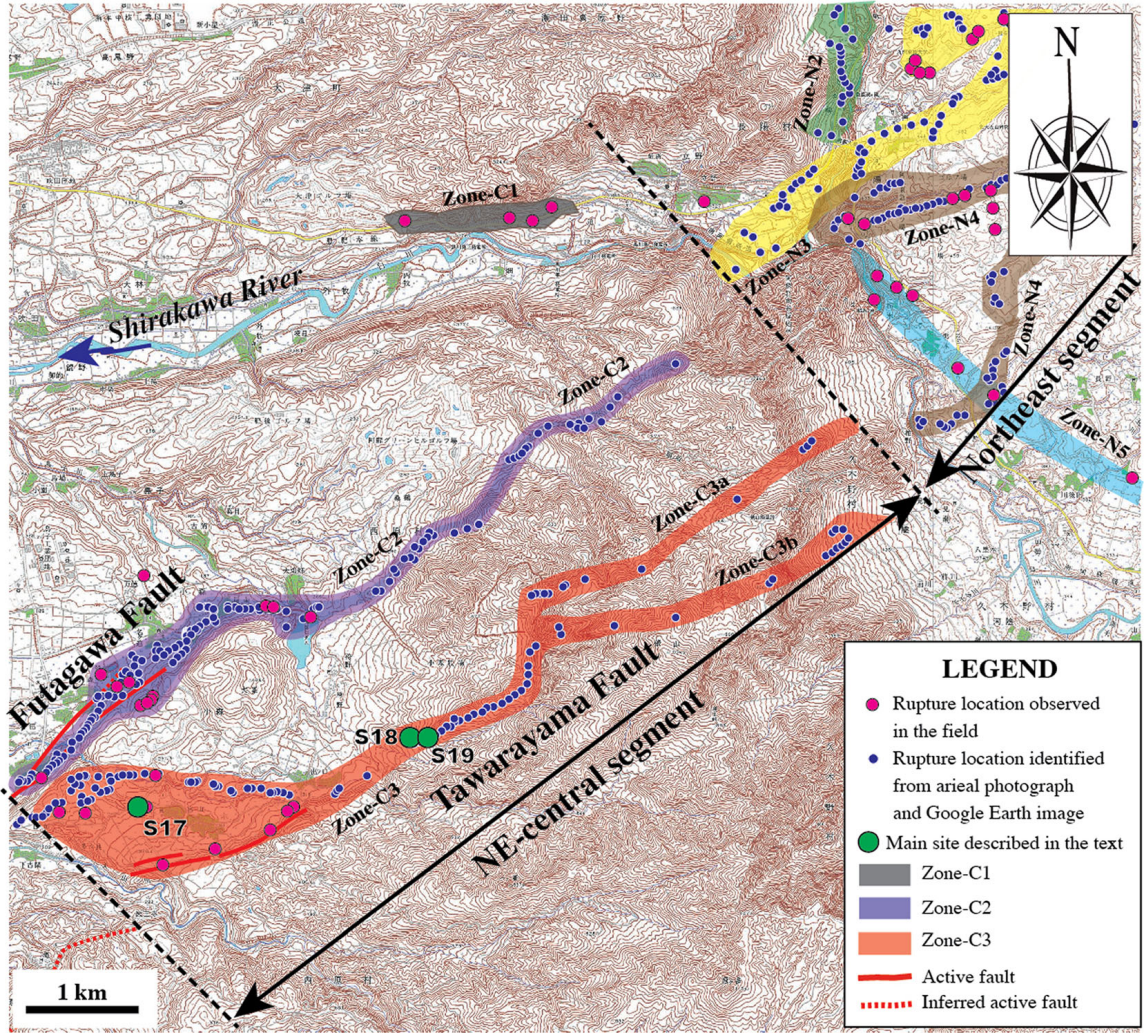
Topographic map showing the distribution of coseismic surface ruptures along the NE-central segment. Active fault data are from RGAFJ (1980, 1991) and Geographical Survey Institute (2001) (using 1:25,000 topographical map released by the Geographical Survey Institute)

The NE-central segment, striking NE-SW, mainly occurred on the southwestern slope of Mount Aso and comprises four subparallel rupture zones (called Zone-C1 to Zone-C3) (Fig. 3). Zone-C1 is distributed in the northern bank of the Shirakawa River, where numerous houses were mostly collapsed. The subrupture zone occurred on the lowlands, comprising mainly of extensional cracks. Zone-C2 is at the northeast extension of the SW-central segment, along the topographic boundary between the Shirakawa River valley and the slope of Mount Aso, where the Futagawa Fault developed. Zone-C3 is on the southwestern slope of Mount Aso, 2–3 km east of Zone-C2, developed along a newly identified fault [called Tawarayama Fault (TF) in Lin et al. (2016)]. The southwestern end section of Zone-C3 occurred along the inferred active right-lateral strike-slip fault (called Idenokuchi Fault) (Watanabe et al. 1979; RGAFJ 1980, 1991), and the northeast part of Zone-C3 is branched into two subparallel zones (Zone-C3a and Zone-C3b) (Fig. 3). It was difficult to access the rupture locations of Zone-C1 and Zone-C3, primarily due to earthquake damage to mountain roads. Therefore, most coseismic surface ruptures in this zone were identified from the high-resolution Google Earth images acquired on 18 April 2016 after the earthquake.

**Fig. 4:**
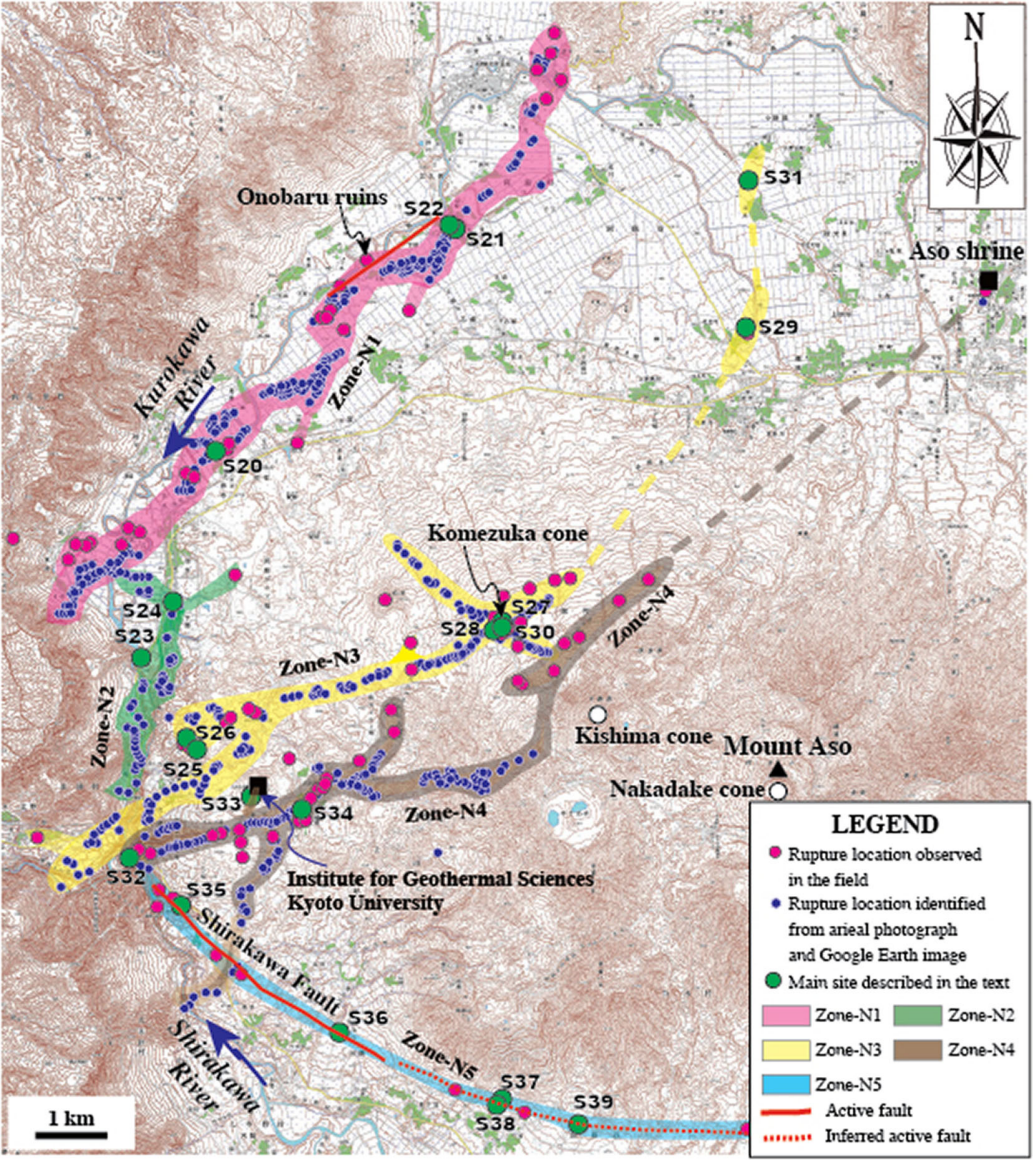
Topographic map showing the distribution of coseismic surface ruptures along the northeast segment inside Aso caldera (using 1:25,000 topographical map released by Geographical Survey Institute). Active faults at the Onobaru site in Zone-N1 is from Sudo and Ikebe (2001) and along Zone-N5 is identified in this study

In contrast to the central and southwest segments, the northeast segment shows a relatively complicated distribution of surface rupture patterns in a wide area around the western and southern sides of Aso caldera. Based on the distribution and deformation features, this segment can be subdivided into five branch rupture zones (called Zone-N1 to Zone-N5), each with a different orientation (Fig. 4). Zone-N1, striking N40–60° E, is mainly composed of normal faults and extensional cracks that form graben structures (see below for details), extending ∼10 km along the northwestern edge of Aso caldera (Fig. 4). The generation of the coseismic graben inside the caldera is interpreted to be caused by the presence of the magma chamber under the caldera that induced an upward pressure, resulting in localized E-W to NNW-SSE extensional stresses (Lin et al. 2016). Zone-N2 occurs along the southwestern edge of Aso caldera, striking N-S, and oblique to Zone-N1. Zone-N3 is the northeastern extension of Zone-C2 of the NE-central segment, which crosscuts the southwestern rim of Aso caldera and Komezuka cone (inside the caldera) with a conjugate geometric pattern of ruptures striking N50–60° E and N50–70° W, respectively (Fig. 4). Surface ruptures are also found in the area around the crater and foot of Komezuka cone in a doughnut-shaped pattern (see below for details). The ruptures of Zone-N1 and Zone-N3 terminated at the northeastern side, near the northern edge of the caldera (Fig. 4). Zone-N4 shows more irregular geometric pattern than Zone-N1, which is locally bended and branched. This zone is subparallel to the general trend of Zone-N3, 0.5–3 km east of Zone-N3, crosscuts the southwest-northwestern side of Aso caldera, bounded by Kishima and Nakadake cones in the east and Komezuka cone in the west, and terminates at the northeastern edge of the caldera (Fig. 4). Rupture in this zone was inferred to terminate near Aso Shrine in the northeast (Fig. 4), which was completely destroyed by the earthquake. Zone-N5 lies on the southern slope of Nakadake cone along the southern edge of the caldera (Fig. 4). Coseismic surface ruptures were observed along a linear scarp striking N70–80° W and dipping south, which developed on alluvial fans formed from southward-flowing drainages in the northern side of the northwestward flowing Shirakawa River (Fig. 4). Topographically, this scarp develops along a topographical boundary between the lowland of the Shirakawa River valley and the mountain slope of Mount Aso.

### Structural features of coseismic surface ruptures

**Fig. 5:**
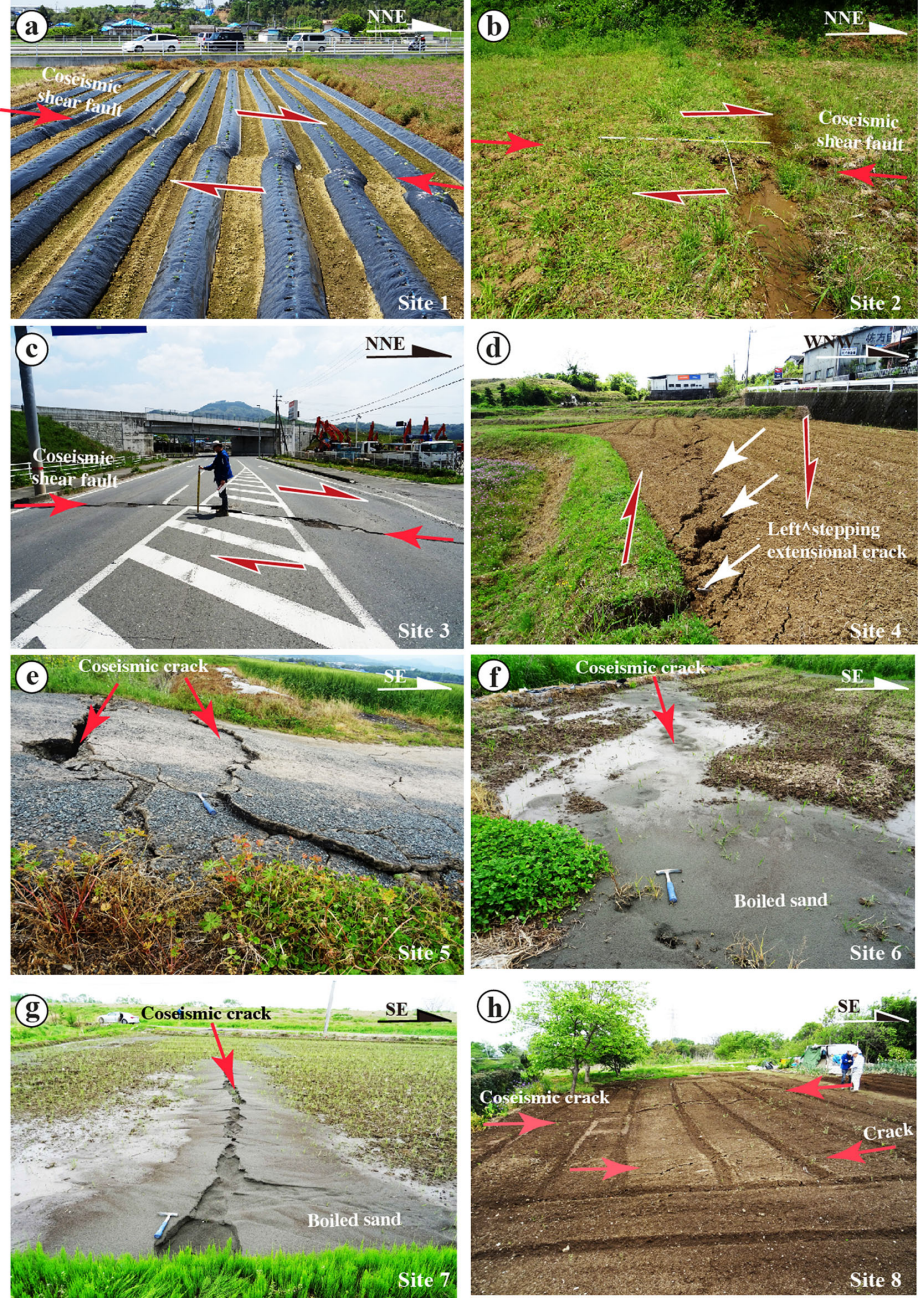
Representative photographs of coseismic surface ruptures along Zone-S1 (**a**–**d**) and Zone-S2 (**e**–**h**) of the southwest segment. **a** Right-lateral displacement of a vegetable field at site 1. **b** Right-lateral displacement of a field path at site 2. **c** Right-lateral displacement of a road at site 3. **d** Left-stepping extensional cracks at site 4. **e** Coseismic cracks at site 5. **f** Liquefaction occurred at site 6. **g** Liquefaction at site 7. **h** Flexural structure occurred at a vegetable field (site 8) on which surface cracks duplicated

**Fig. 6:**
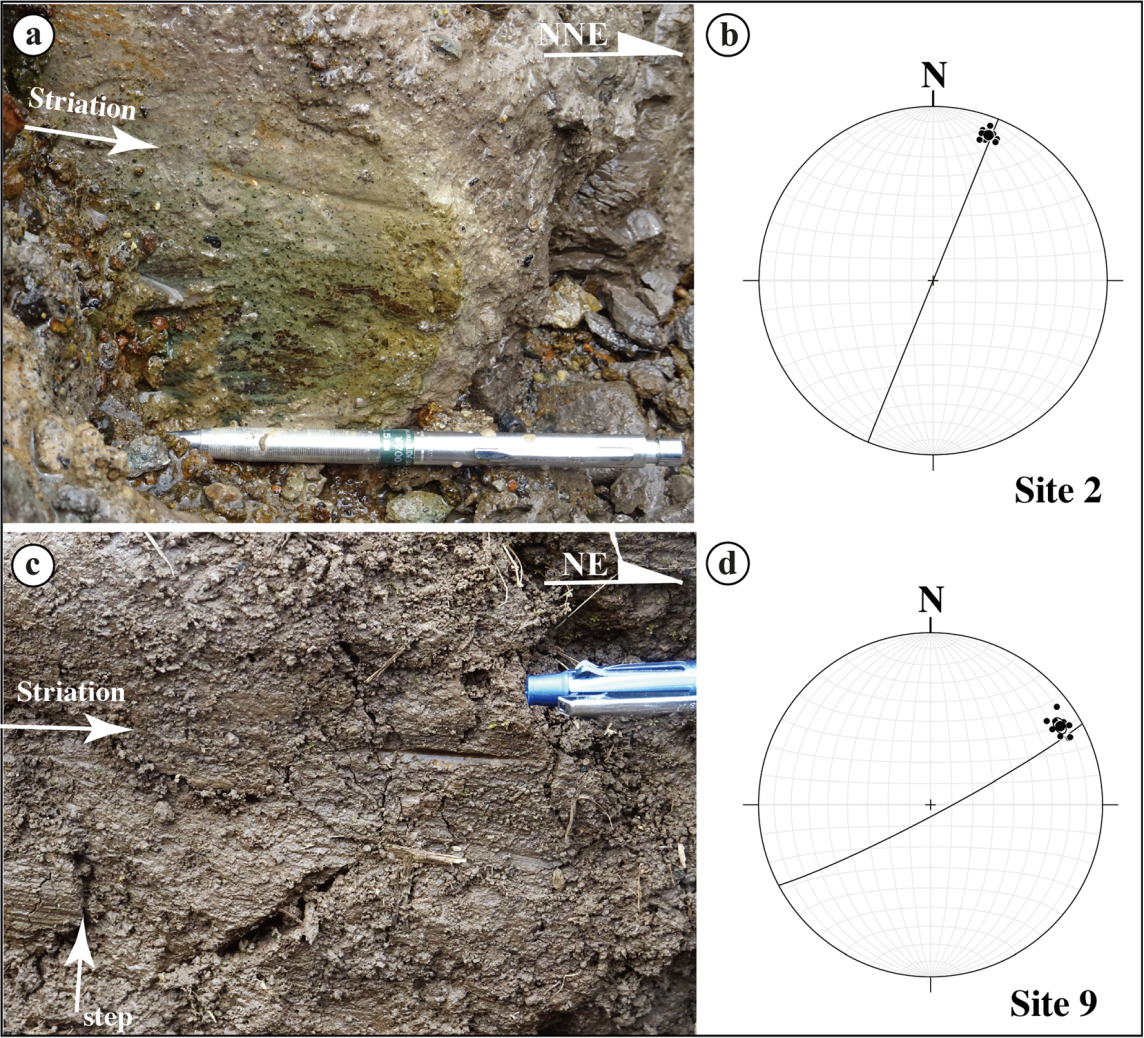
Photographs (**a**, **c**) and stereographic projections (**b**, **d**) show the orientations of the fault planes and striations. **a** Striations on the main fault plane at site 2. **b** Stereographic projection of **a**. **c** Striations on the main fault plane at site 9. **d** Stereographic projection of **c**

Field investigations show that coseismic surface ruptures created different structural features in each of the four principal rupture segments. Zone-S1 is mainly composed of distinct strike-slip faults, left-stepping echelon cracks, and mole tracks that occurred mostly along the main segment of the Hinagu Fault (Fig. 5a–d). Distinct strike-slip faults striking N10–20° E and dipping 75–90° NW, subparallel to the general trend of the rupture zone, are dominated by right-lateral strike-slip movement (Fig. 5a–c). Horizontal slickenside striations observed on shear fault planes, marked by parallel lineations with some grooves and steps in unconsolidated clay, also show strike-slip-dominated movement (Fig. 6a, b). In contrast, Zone-S2 is mainly composed of extensional cracks and flexural structures (Fig. 5e–h). The surface cracks are distributed over a wide area, and no distinct offset is observed. Liquefaction of sandy material occurred along the extensional cracks, in lowland areas near river channels, and was characterized by boiled sandy material along extensional cracks (Fig. 5f, g). Flexural structures formed in a field of vegetables as a waveform pattern, on which the extensional cracks duplicated (Fig. 5h).

**Fig. 7:**
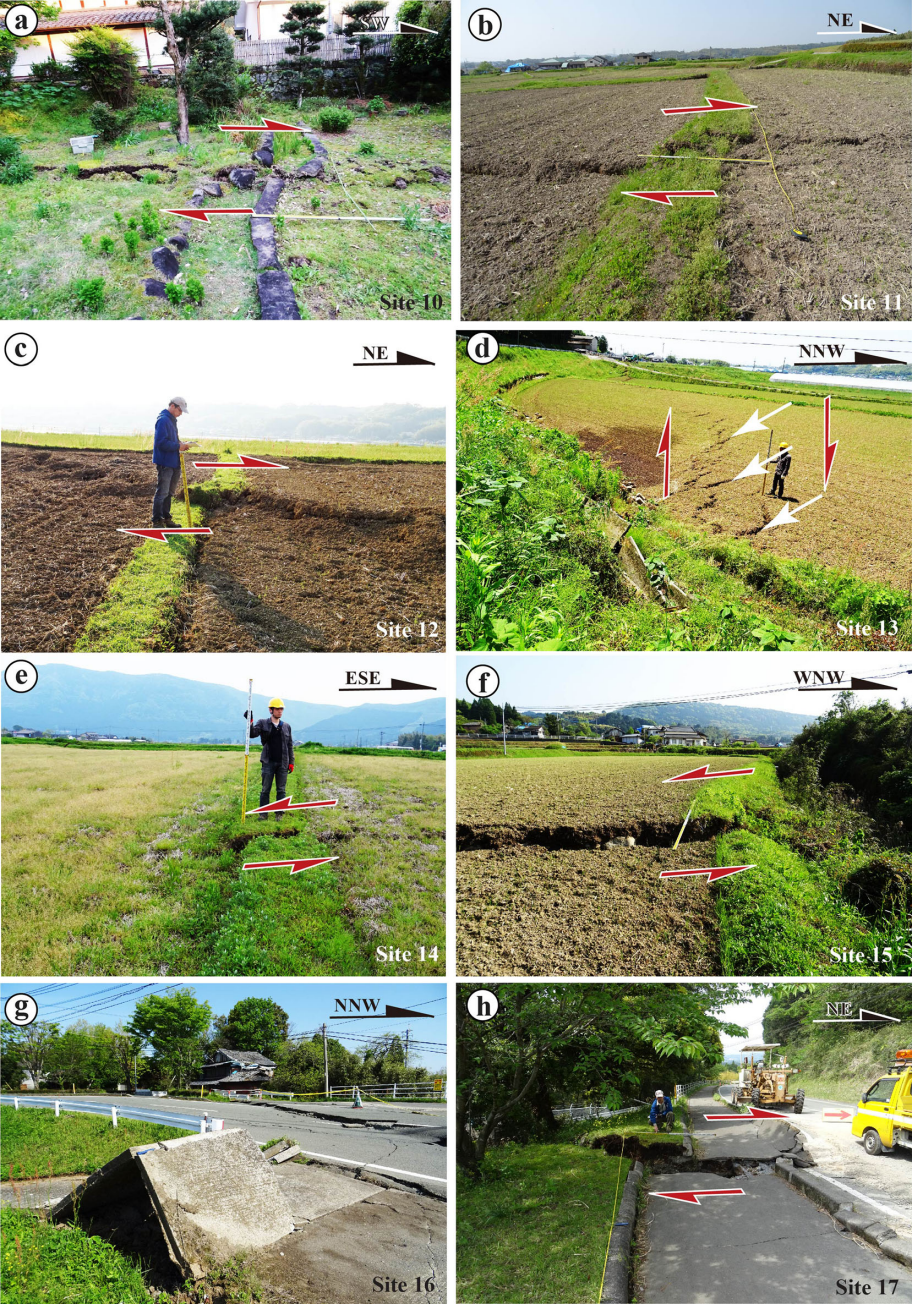
Representative photographs of coseismic surface ruptures in the SW-central segment. **a**–**d** Right-lateral displacements at site 10 (**a**), site 11 (**b**), site 12 (**c**), and site 13 (**d**), respectively. **e** Left-stepping echelon cracks occurred at site 14. **f**, **g** Left-lateral strike-slip displacement at site 15 (**f**) and site 16 (**g**). **h** Mole track occurred on a concrete road at site 17

**Fig. 8:**
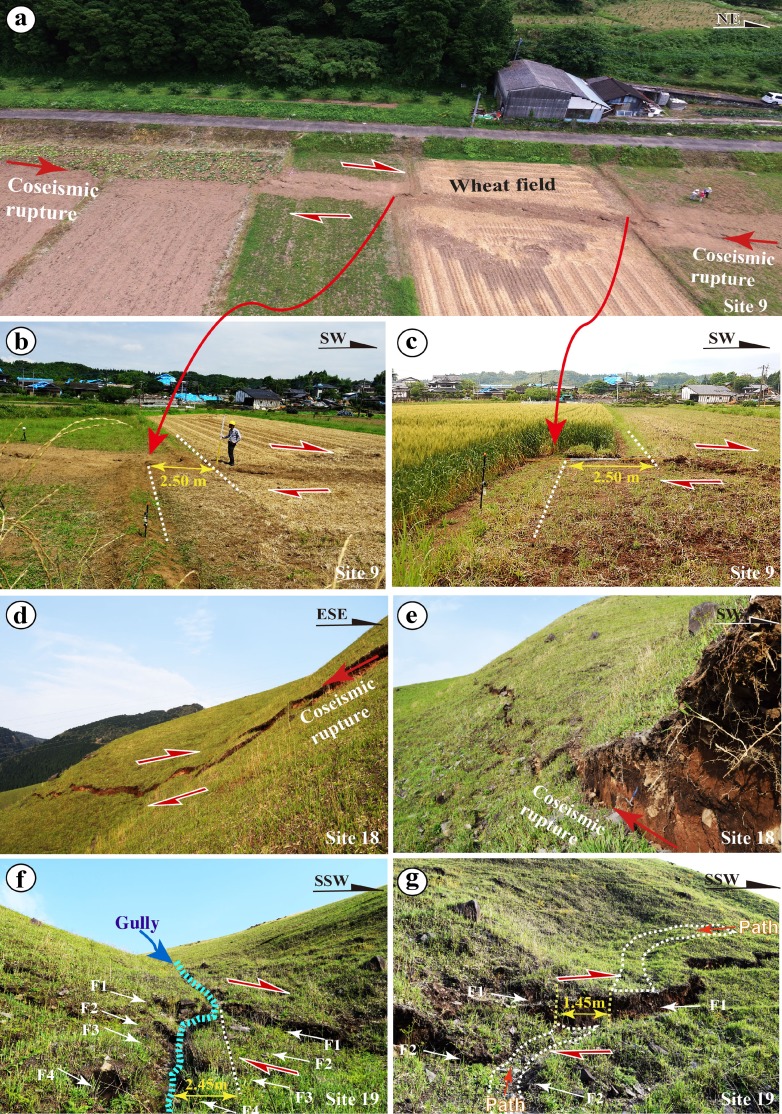
Representative photographs showing the deformation features of coseismic surface ruptures in SW-central segment at site 9 (**a**–**c**) and Zone-C3 of NE-central segment on the mountain slope where a right-lateral displacement of up to 2.45 m was observed (**d**–**g**). **a** Photograph taken by a drone. **b**, **c** Right-lateral strike-slip displacement at both northeast and southwest sides of the wheat field shown in **a**, where the field paths were offset by 2.50 and 2.45 m, respectively. **d**, **e** Coseismic surface ruptures occurred on the mountain slopes at site 18. **f** A gully was right-laterally offset by 2.45 m with a vertical component of 0.5 m at site 19. **g** A mountain path was right-laterally offset by 1.45 m with a vertical component of 0.3 m at a location near site 19

The SW-central and NE-central segments are mainly characterized by distinct strike-slip faults, extensional cracks, and mole tracks (Figs. 7 and 8). The strike-slip faults mostly follow the NE-SW trend of the Futagawa Fault along the SW-central segment and in Zone-C2 of the NE-central segment, along which distinct right-lateral strike-slip displacements are observed (Figs. 7 and 8a–c). A typical example of right-lateral strike-slip shear faults is observed at site 9, where a maximum offset of ∼2.5 m was measured (Fig. 8a–c). The horizontal offsets are also indicated by slickenside striations developed on strike-slip fault planes at this site, marked by parallel lineations with some grooves in unconsolidated clay (Fig. 6c, d). Locally, some WNW-ESE-striking shear faults are also observed in the NE segment, along which left-lateral strike-slip displacements are observed (Fig. 7f, g). These form a typical conjugate fault pattern with the NE-SW-striking shear faults. The extensional cracks commonly show left-stepping echelon patterns also that indicate a right-lateral strike-slip sense of shear and are widespread along the NE-striking surface rupture zone (Fig. 7e). Mole tracks, ranging from 20 cm to 1 m high, are found in the area between two adjacent cracks as those observed at site 17, and mostly occur in asphalt and concrete roads (Fig. 7h). This combination of deformation features of coseismic surface ruptures and slickensides on fault planes reveals that the host fault experienced predominantly right-lateral strike-slip surface motion in the southwest-central segment. Zone-C3 on the mountain slope is mainly composed of right-lateral strike-slip shear faults with distinct normal offset component (Fig. 8d–g). A large right-lateral strike-slip offset of 2.45 m is observed at site 19, where a small gully was dextrally offset by three parallel strike-slip faults (Fig. 8f). Whether or not these strike-slip faults occur along the pre-existing active fault remains unclear, due to the lack of geological data, and therefore, further work is needed to resolve this issue.

**Fig. 9:**
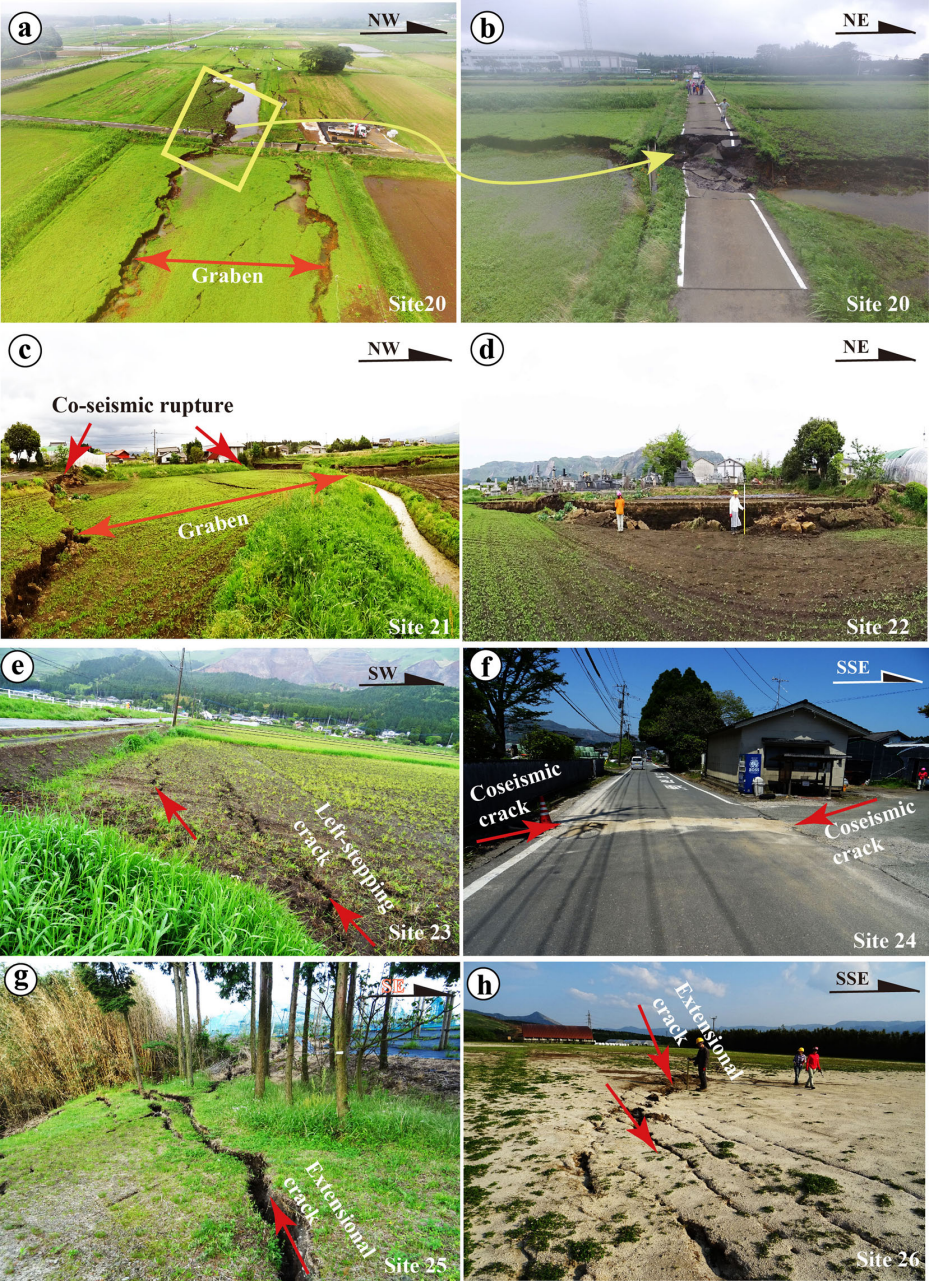
Representative photographs showing the deformation features of coseismic surface ruptures along Zone-N1 (**a**–**d**), Zone-N2 (**e**, **f**), and Zone-N3 of the northeast segment (**g**, **h**). **a**–**d** Coseismic surface ruptures that formed graben structures in Zone-N1 at site 20 (**a**, b), site 21 (**c**), and site 22 (**d**). **a**, **b** Photographs taken by a drone showing a graben structure where maximum vertical offset of up to 1.75 m was observed (**b**). **c**, **d** A graben structure observed at site 21 where a vertical offset of 1.3 m was observed at site 22 (**d**). **e** Left-stepping cracks of Zone-N2 at site 23. **f** Coseismic extensional cracks of Zone-N2 at site 24. **g**, **h** Coseismic extensional cracks of Zone-N3 at sites 25–26

**Fig. 10:**
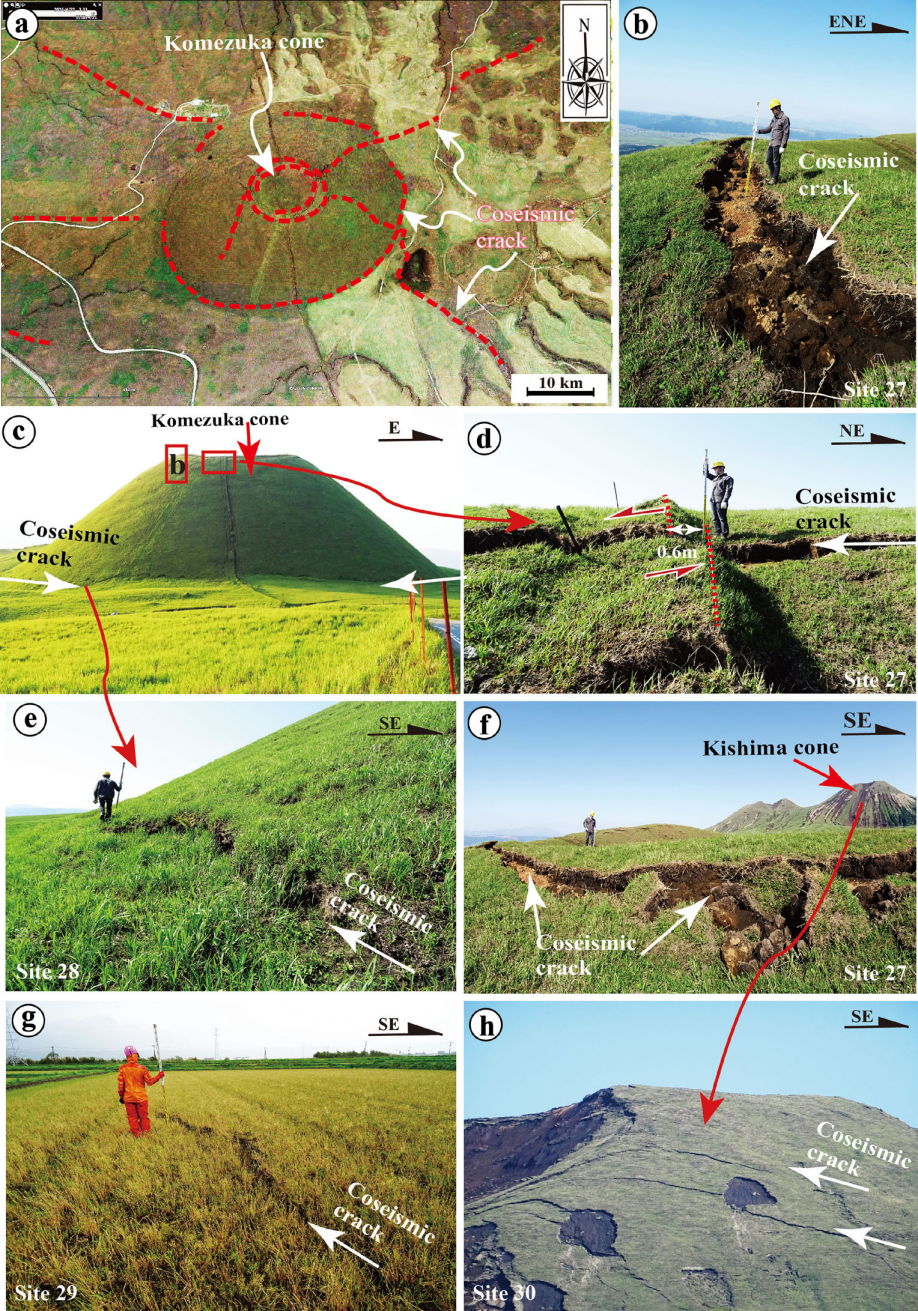
Representative photographs showing deformation features of coseismic surface ruptures of Zone-N3 along the northeast segment in the area around Komezuka cone. **a** Google Earth image acquired on 18 April 2016, showing distribution features of coseismic surface ruptures crosscut Komezuka cone. **b** Coseismic extensional cracks on the western rim of Komezuka cone (site 27). **c** Northward view of Komezuka cone. **d** Coseismic extensional cracks on the southern rim of the cone (site 27). **e** Coseismic cracks on the southwestern foot of the cone (site 28), where the Komezuka cone is uplift by 90 cm. **f** Coseismic extensional cracks on the northwestern rim of Komezuka cone (site 27). **g** Coseismic cracks at site 29 in the northeastern end area of Zone-N3. **h** Coseismic extensional cracks on the northwestern slope of the Kishima cone viewed from the top of Komezuka cone (site 31) (see Fig. 4 for detail location)

**Fig. 11:**
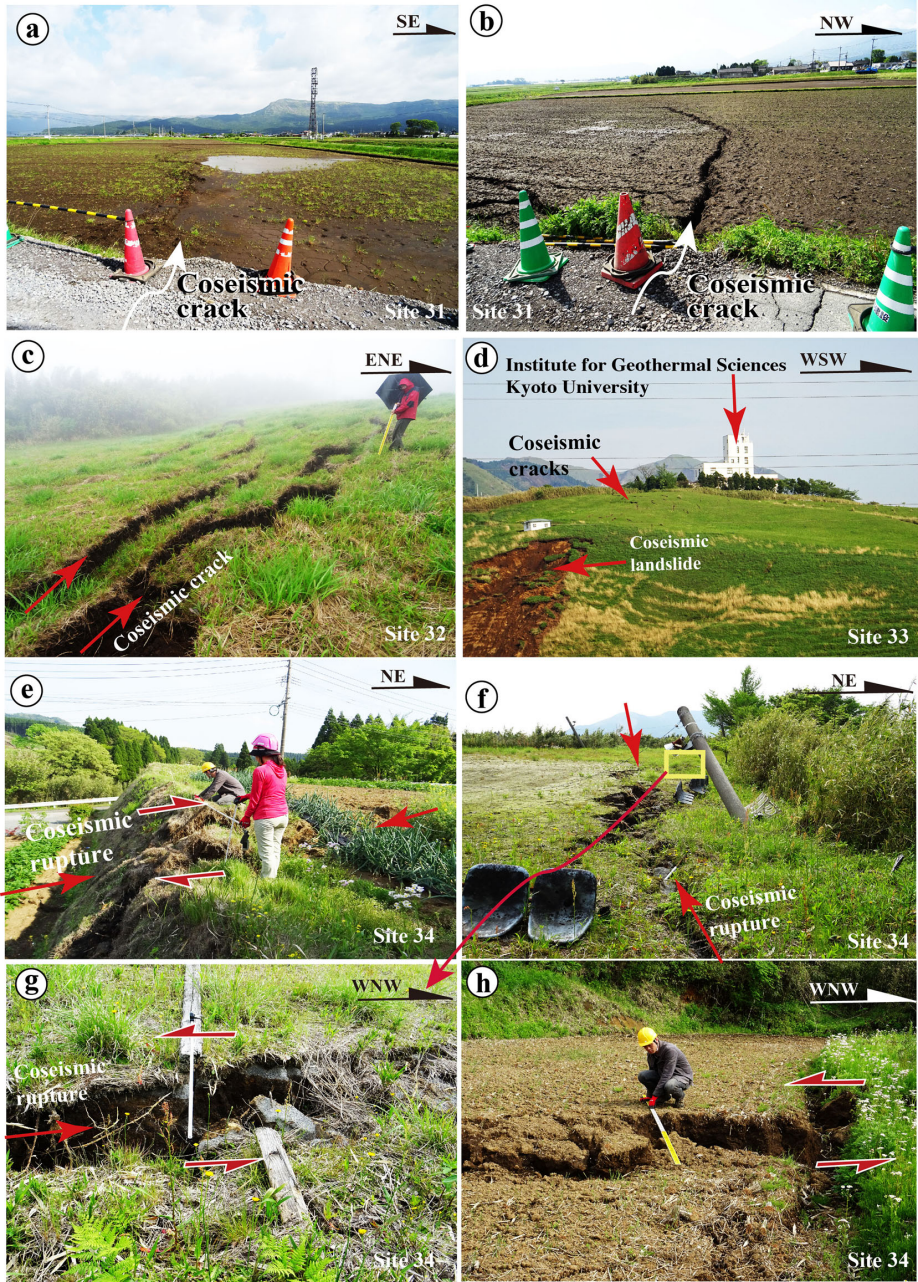
Representative photographs showing deformation features of coseismic surface ruptures of Zone-N3 (**a**, **b**) and Zone-N4 (**c**–**h**) along the northeast segment. **a**, **b** Coseismic cracks at the southwest end of Zone-N3. **c** Coseismic cracks at site 34. **d** Coseismic ruptures and coseismic landslide occurred along a mountain slope at the Institute for Geothermal Sciences, Kyoto University (site 32). **e**, **f** Coseismic surface ruptures at site 33, showing right-lateral displacements (**e**) and left-lateral displacements (**g**, **h**)

**Fig. 12:**
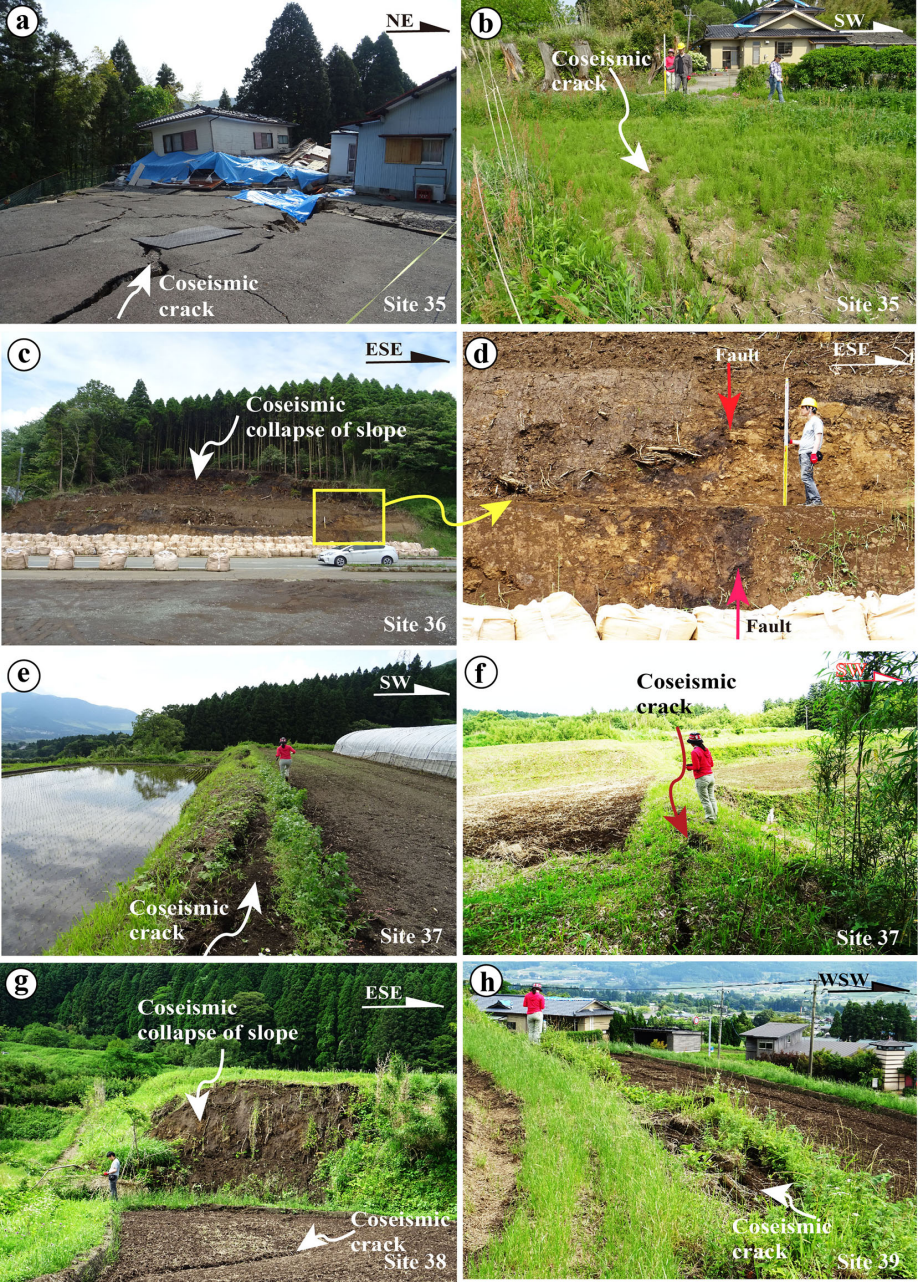
Representative photographs showing deformation features of coseismic surface ruptures of Zone-N5 along the northeast segment. **a** Collapsed houses on the coseismic surface ruptures at site 34. **b** Coseismic cracks at site 34. **c**, **d** Fault outcrop at an earthquake-caused collapse of a mountain slope at site 35. **e**, **f** Coseismic cracks at site 36. **g**, **h** Coseismic surface ruptures occurred along fault scarp at site 37 (**g**) and site 38 (**h**)

In contrast to the southwest-central segments, the northeast segment is dominated by normal faults, extensional cracks that form graben structures, and some shear faults with horizontal displacement sense (Figs. 9, 10, 11, and 12). Zone-N1 is characterized by normal faults striking N40–60° E with dip angles of 75–90°, forming a typical graben structure that varies in width from 20 to 100 m (typically 30–50 m), with a vertical offset up to 1.75 m on both sides of the graben (Fig. 9a–d). Zone-N2 is mainly composed of extensional cracks, which generally occur as an array of parallel to subparallel cracks without distinct echelon geometric patterns, in contrast to those observed along the central segments (Fig. 9e–f). Zone-N3 is mainly composed of extensional cracks with opening widths up to 50 cm and distinct shear faults striking N50–60° E and N60–70° W, along which both right-lateral strike-slip and left-lateral strike-slip displacements up to 60 cm are observed along the NE- and NW-striking faults, respectively (Figs. 9g, h and 10a–g). Both the crater and the cone are uplifted 30 cm relative to the slope and 50 cm relative to the area surrounding Komezuka cone (Fig. 10a–f). This observation indicates that the NE- and NW-striking rupture zones crosscut the volcano cone by conjugate ruptures under E-W compressive stress, coinciding with the direction revealed by seismic data and geodesic measurements (Geological Survey of Japan, AIST 2016). The rupture of this zone terminates in the northern edge of Aso caldera (Fig. 4). Zone-N4 is subparallel to the general trend of Zone-N3, crosscuts the western slope of Kishima cone, and terminates at the northeastern edge of Aso caldera, where the Aso Shrine was completely collapsed (Figs. 11c–g and 12a, b). Zone-N5 developed along a linear scarp developed along the topographical boundary between the southern slope of Mount Aso and the lowland of Shirakawa River valley (Figs. 4 and 12c–h), where a fault outcrop was observed at an earthquake-caused collapsed slope (Fig. 12c, d). At this outcrop, unconsolidated deposits, including volcanic deposits and dark surface soil layers, are vertically offset (Fig. 12d). These observations indicate that the coseismic surface ruptures occurred on an active fault scarp developed on the alluvial fans. We are still working on this fault scarp to understand the recent activity, including the most recent faulting timing and the relevant structural features of this newly identified active fault inside Aso caldera.

The ground deformation features and distribution patterns of the northeast segment of the coseismic surface rupture zone observed in this study reveal that the SW-NW rim of Aso caldera and Komezuka and Kishima cones have been crosscut by coseismic ruptures and that the coseismic surface rupturing propagation stopped inside Aso caldera.

### Coseismic displacements

**Fig. 13:**
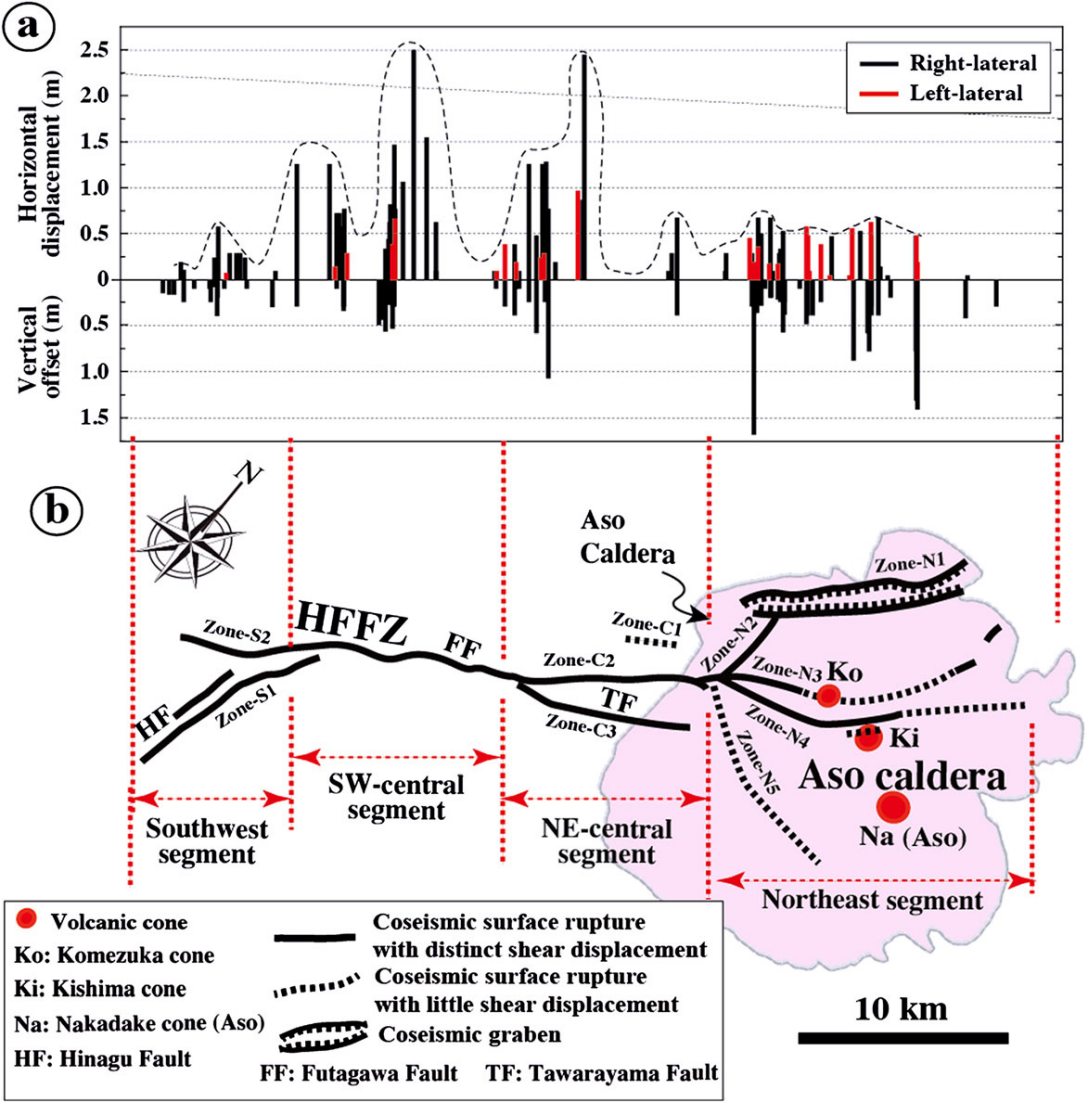
Displacements and distribution and rupture traces of coseismic surface rupture zone (modified from Lin et al. 2016). **a** Distribution of coseismic displacements measured in-site. **b** Map showing the distribution of the coseismic surface rupture zone

Field measurements of coseismic displacements are plotted in Fig. 13, representing samples taken at 148 sites (Table S1). The maximum right-lateral displacements observed at sites 9 and 19 in the SW- and NE-central segments, respectively, are up to 2.45–2.50 m (Fig. 8a–c, f). At site 9, the deformation features of surface ruptures and slickenside striations observed in the wheat field indicate a pure right-lateral strike-slip movement with little vertical component along the left-stepping echelon shear faults (Figs. 6c, d and 8a–c). On the mountain slope, right-lateral strike-slip displacements on the mountain slope are often accompanied by distinct normal fault offset component along the coseismic shear faults as that observed at site 19. In both the SW- and NE-central segments, left-lateral displacements were observed locally along NW-striking shear faults (Fig. 13) as that observed at sites 15 and 16 (Fig. 7f, g), in which the maximum offset amount is 0.9 m. In contrast, the northern segment inside Aso caldera is dominated by vertical displacement of up to 1.75 m with a minor horizontal component in both the left-lateral and right-lateral strike-slip faults that form conjugate fault structures (Fig. 11c–h, Table S1).

## Discussion

### Relationship between coseismic surface rupture and pre-existing active faults

The structural and geometric characteristics of coseismic surface ruptures not only reflect the surface rupture morphology but also the structure at depth and the pre-existing tectonic environment of a seismogenic fault (e.g., Yeats et al. 1997; Lin et al. 2002, 2009). Field investigations carried out in this study during the past half year after the main shock demonstrate that the coseismic surface rupture of the Kumamoto earthquake occurred mostly along the main traces of the pre-existing Hinagu–Futagawa fault zone in the southwest and central segments (Figs. 2 and 3). The focal mechanism solutions of the *M*_w_ 6.2 (Mj 6.5) foreshock and the *M*_w_ 7.1 (Mj 7.3) main shock show predominantly strike-slip motion on a fault striking NE-SW and dipping northwest at 60–85°, with a compressional axis oriented E-W (Fig. 1b; Japan Meteorological Agency 2016a; National Research Institute for Earth Science and Disaster Prevention 2016). Seismic inversion results reveal that the southwest segment of the source fault is dominated by right-lateral strike-slip displacement, but normal-slip components are accompanied on the fault plane of the northeast segment of seismogenic fault (Asono and Iwata 2016). These seismic results are consistent with what I observed in the field. Recent trench investigations reveal that at least two to four morphogenic earthquakes occurred in the late Holocene on both the Hinagu and Futagawa faults, at site 2 and site 9, respectively, where the coseismic surface ruptures with distinct dextral displacement of up to ∼62 cm (site 2) and 2.5 m (site 9) occurred, indicating that the active faults of the HFFZ are currently active as seismogenic faults of large earthquakes during the Holocene (Lin et al. 2017).

**Fig. 14:**
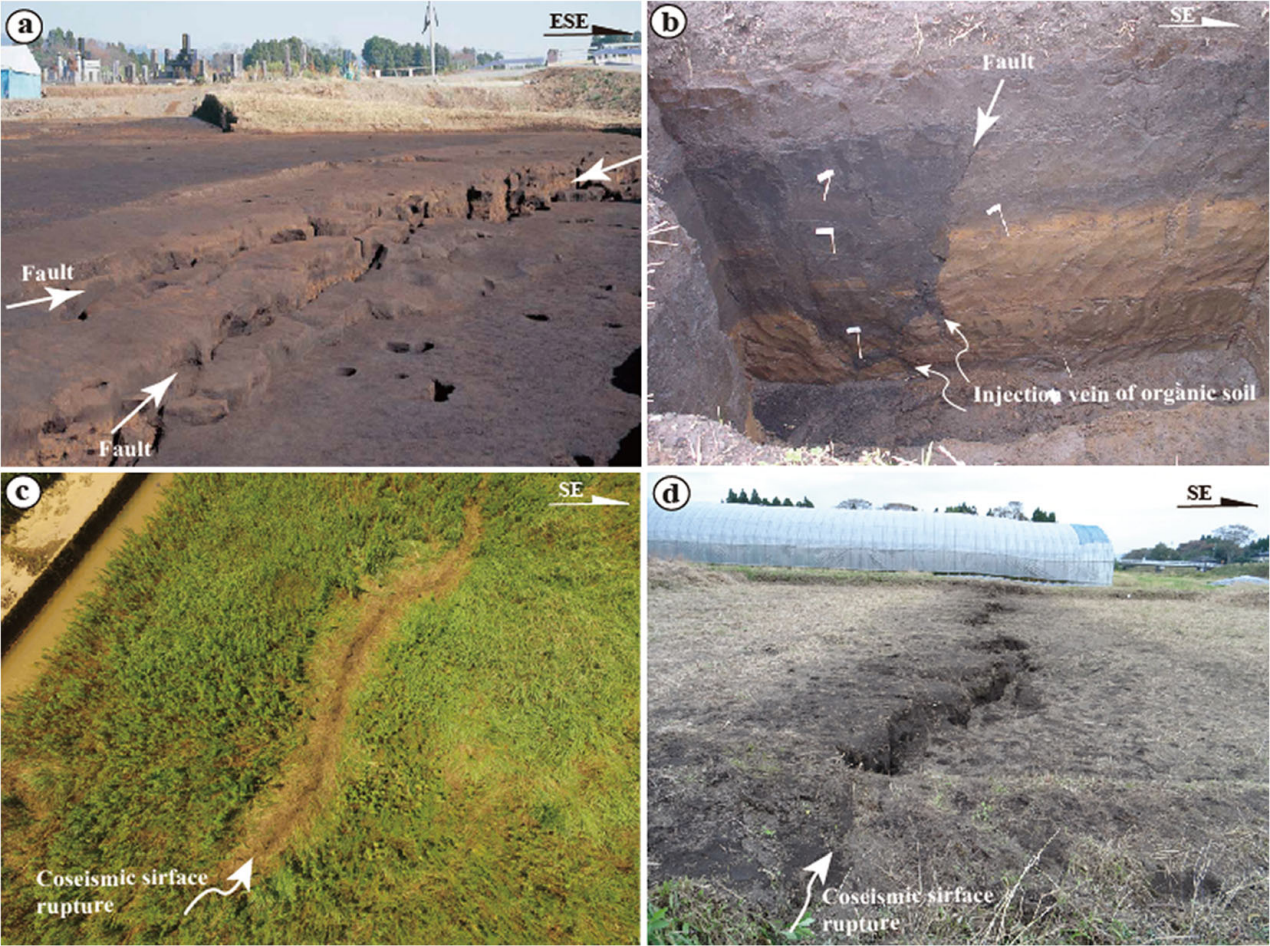
Photographs showing pre-existing active fault (**a**, **b**) and deformation features of coseismic surface ruptures of Zone-N1 observed at a location, 1 km southwest of site 22. **a** A graben structure exposed at a Yayoi ruin site [photo cited from Education Committee of Kumamoto Prefecture (2010)]. **b** Normal fault structure exposed at a construction section, where the dark organic soil materials are injected in the yellowish volcanic deposits. Photo courtesy of Dr. Y. Sudo. **c** Coseismic surface rupture superimposed on the graben structure of **a**. **d** Coseismic surface rupture occurred on the southeast extension of graben structure, ∼70 m from the Yayoi ruin site shown in **a**. Note the southwestern side was uplift ∼30 cm

In contrast, the focal mechanism solutions of the *M*_w_ 5.3 (Mj 5.5) and *M*_w_ 5.7 (Mj 5.9) aftershocks, 20–25 min after the main shock in the central-northeast segment of the rupture, show predominantly normal faulting (Fig. 2b), consistent with the graben structures of coseismic ruptures observed in the field. A previous study reported that local ruins (Onobaru ruins; see Fig. 4 for detail location) from the Yayoi period (ca. 300 BC–300 AD) were destroyed by a graben structure formed by a faulting event that occurred ∼2000 years BP (Fig. 14a, Education Committee of Kumamoto Prefecture 2010). Sudo and Ikebe (2001) reported that (1) an active fault striking N60° E and dipping 60° to the southeast was exposed during road construction at a location near the Yayoi site close to site 22 (Fig. 14b) and (2) the fault that cuts the Aso volcanic deposits formed in the past 1000–15,000 years. The most recent faulting event is inferred to have occurred in the past ∼1000–2000 years (Sudo and Ikebe 2001), consistent with that observed in the Yayoi ruins. Field investigations in this study also show that the 2016 coseismic surface ruptures superimposed on the graben structures exposed in the ruin site with a distinct vertical offset of up to 50 cm (Fig. 14c, d). Our field observations reveal that the coseismic surface ruptures of Zone-N1 almost duplicated the pre-existing active fault as shown in Fig. 4. The coseismic surface ruptures of the Zone-C5 striking WNW-ESW also duplicated a pre-existing fault where the surface soil and young volcanic deposits are offset as observed at site 35 (Fig. 12c, d). These geological and archaeological data indicate that the large earthquakes occurred repeatedly inside the caldera on the pre-existing active faults.

**Fig. 15:**
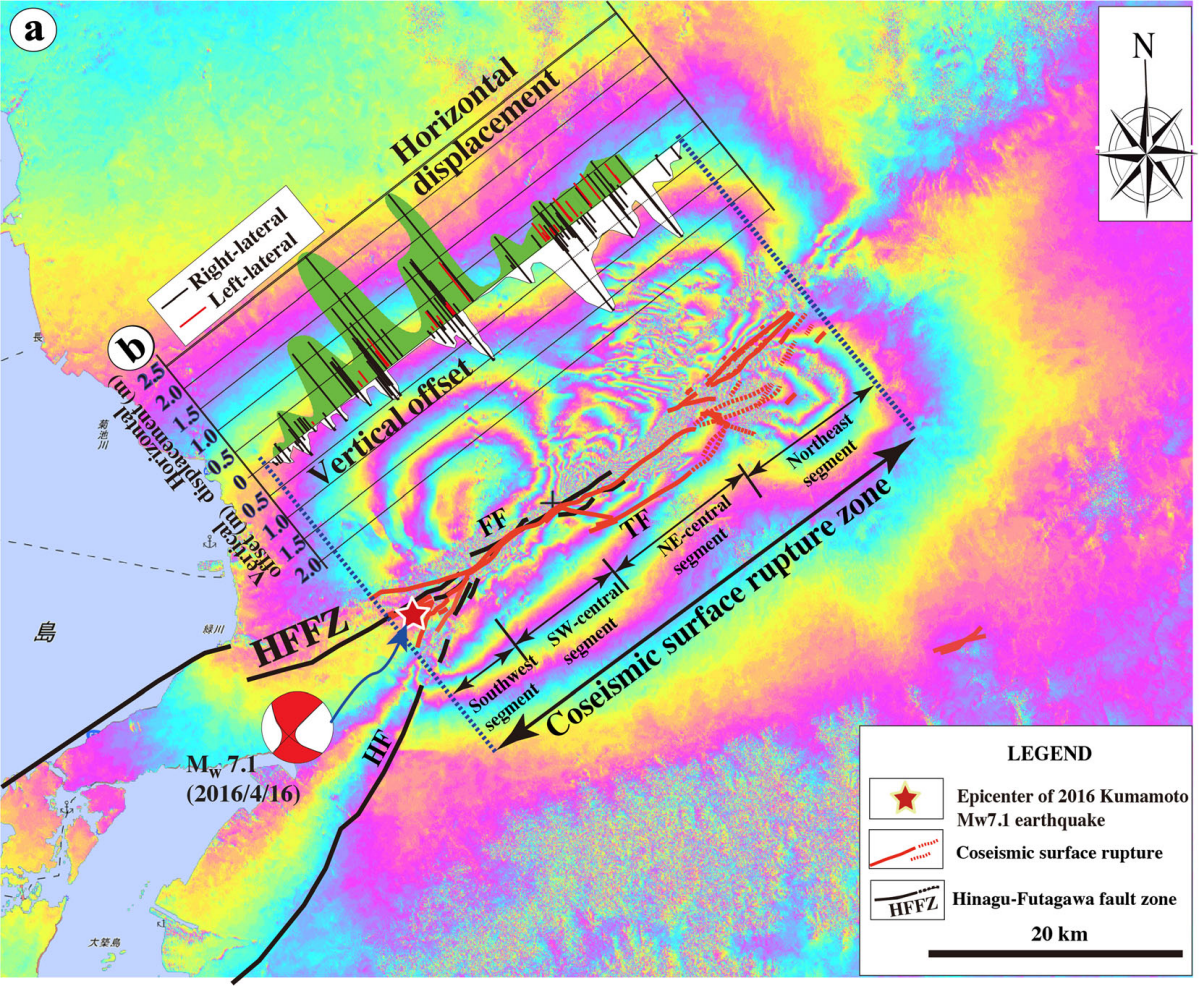
InSAR image (**a**) generated from PALSAR-2 data acquired on 16 January 2016 and 20 April 2016 and displacement distribution along the coseismic surface rupture zone showing the deformation features of ground surface caused by the 2016 Kumamoto earthquake [modified from the Geospatial Information Authority of Japan (2016)]. *Color fringes* are contours of equal ground displacement along the line of sight of the satellite. One full-color cycle represents ∼12 cm surface displacement parallel to the line of sight. **a** The coseismic surface displacement zone was up to ∼40 km in length. *HF* Hinagu Fault, *FF*: Futagawa Fault, *TF* Tawarayama Fault. **b** The field-measured offset distribution along the coseismic surface rupture zone is shown for comparison. Note that discrete surface ruptures were restricted to a ∼40-km-long surface rupture zone

InSAR data revealed that the total ground rupture length of the seismogenic fault was ∼40 km with a maximum offset of up to >1.0 m (Fig. 15). The InSAR analysis shows that (i) the distinct ground deformation occurred in two sections, one along the southwest and SW-central segments that are dominated by strike-slip movement, and the other along the northeast segment of the coseismic surface rupture zone that is dominated by normal faults as graben structures inside Aso caldera (Fig. 15). InSAR data also show that the deformation zones developed inside Aso caldera show a complex geometry where the rupture zones are concentrated in five linear disturbed zones with different orientations which are consistent with our field observations (Figs. 13a and 15). Analytical results of InSAR data based on offset tracking technique showed the different distribution features of 3D displacements around the epicenters of three *M*_w_ >6 earthquakes including two foreshocks of *M*_w_ 6.2 and *M*_w_ 6.0 which occurred on 14 April 2016 and main shock *M*_w_ 7.1 on 16 April 2016 along the different segments (Himematsu and Furuya 2016). The inferred slip distributions at different segments indicate that while the right-lateral strike-slip displacement was dominated at the shallower depths of F1 (Futagawa Fault) and F2 (Hinagu Fault), only normal faulting is significant at greater depth of F3 (Tawarayama Fault identified in this study), suggesting the occurrence of slip partitioning during the Kumamoto earthquake sequence (Himematsu and Furuya 2016). This analytical result is also consistent with the field observations that the Hinagu and Futagawa faults are dominated by right-lateral strike-slip displacements and that the distinct normal slip component occurred along the newly identified Tawarayama Fault as shown in Figs. 13 and 15.

The geological and archaeologic evidence and seismic and InSAR data shown in this study demonstrate that the distribution patterns and coseismic displacements and structural features of coseismic surface ruptures are controlled by pre-existing active Hinagu–Futagawa fault zone and active faults inside Aso caldera.

### Seismotectonic implications of coseismic rupture zones

Seismic inversion results show that the coseismic rupture initiated at the jog area between the Hinagu and Futagawa faults, propagated northeastward through Aso caldera, and terminated within the caldera (Koketsu et al. 2016; National Research Institute for Earth Science and Disaster Prevention 2016; Yagi et al. 2016). The field investigations in this study demonstrate that the coseismic surface rupture zone extended from the Futagawa Fault, crosscut Aso caldera for >12 km, and terminated at the NNE edge of the caldera (Lin et al. 2016). However, there remains a question of whether or not the coseismic surface ruptures inside the caldera reflect the upper crustal structure beneath the caldera.

Geophysical observations and analyses of seismic waves reveal that the crustal structures beneath Aso caldera are characterized by a zone of ascending magma with a lower bound ∼3 km below sea level within the caldera and ∼10 km below sea level outside the caldera (Sudo 1988, 1991; Okubo et al. 1989, Okubo and Shibuya 1993; Tsutsui and Sudo 2004; Unglert et al. 2011). Analysis of seismic waves reveals that the low-velocity region (magma chamber) located in the area between the central cone (Nakadake) and the northern rim extends downward from 6-km depth (Sudo 1988). The Futagawa Fault extends to Zone-N3 and Zone-N4, at which the main shock crosscut Komezuka and Kishima cones but stopped at the northeastern edge of the caldera (Figs. 1 and 4). The seismic inversion results also show that up to 1–2 m of fault slip occurred at shallow depths (<6 km) along the seismogenic fault inside Aso caldera, but no distinct slip occurred along the fault plane at >6 km under the caldera (Koketsu et al. 2016; Kubo et al. 2016). The field observations in this study also show that the coseismic surface ruptures are dominated by normal faulting with a maximum vertical offset of up to 1.75 m. These seismic inversion results and field observations demonstrate that the seismogenic fault rupture propagated to the northeastern edge near the surface, where the surface ruptures were observed in the field but stopped at the magma chamber under the caldera at a depth of >3 km. It follows from first principles that neither faults or fractures can develop in a magma chamber if the magma is in a liquid state (Lin et al. 2016). Accordingly, I conclude that Aso’s magma chamber played an important role in stopping the seismogenic rupture as it propagated across the caldera. The 2016 *M*_w_ 7.1 (Mj 7.3) Kumamoto earthquake provided a rare opportunity to study seismotectonics in a volcanic region including the relationship between seismogenic fault processes and crustal structures beneath the Aso volcano cluster, Kyushu Island, southwest Japan.

## Conclusions

Based on the results of field investigations following the 2016 Kumamoto earthquake and considering the discussion above, I arrived at the following conclusions.

1. Geological and seismic data indicate that the pre-existing Hinagu–Futagawa fault zone triggered the 2016 Kumamoto earthquake and controlled the spatial distribution of coseismic surface ruptures.
2. The 2016 Mj 7.3 (*M*_w_ 7.1) Kumamoto (Japan) earthquake produced a ∼40-km-long surface rupture zone, which occurred mostly along the NE-SW-striking Hinagu–Futagawa fault zone in the central and southwest segments, but ruptured newly identified faults inside Aso caldera.
3. The surface displacements of the central and southwest segments were dominated by right-lateral strike-slip motion ranging from several centimeters to 2.5 m with a secondary normal faulting component. In contrast, the coseismic surface ruptures to the northeast were dominated by normal faulting with vertical offsets up to 1.75 m, which formed graben structures inside Aso caldera.
4. The coseismic rupture initiated from the south and propagated northeastward throughout Aso caldera, and terminated within the caldera.

## Electronic supplementary material

Supplementary Table S1 Main locations of the Kumamoto coseismic surface rupture zone, where co-seismic offset amounts were measured in-site. (DOCX 156 kb)
