# Supplementary material for: Structural features and seismotectonic implications of coseismic surface ruptures produced by the 2016 Mw 7.1 Kumamoto earthquake
Source: J Seismol. 2017 Mar 21;21(5):1079–100. doi: 10.1007/s10950-017-9653-5 (PMC5563349; doi:10.1007/s10950-017-9653-5)
Supplement: Supplementary file 1 — Main locations of the Kumamoto coseismic surface rupture zone, where co-seismic offset amounts were measured in-site. (DOCX 156 kb) [file 10950_2017_9653_MOESM1_ESM.docx]

| **Supplementary Table S1.** Main locations of the Kumamoto coseismic surface rupture zone, where co-seismic offset amounts were  measured in-site. | | | | | | | | | | |
| --- | --- | --- | --- | --- | --- | --- | --- | --- | --- | --- |
| **Seg.** | **No.** | **Latitude** | **Longitude** | **Altitude (m)** | **V (m)** | **H (m)** | | **Surface marker** | | **Figure site** |
| S1 | 19 | 32.75823 | 130.81298 | 5.5 | 0.20-0.30 |  |  | Road | |  |
| S1 | 20 | 32.75828 | 130.81361 | 8.4 |  | R | 0.10 | Road | |  |
| S1 | 21 | 32.75832 | 130.81409 | 7.1 |  |  |  | Road | |  |
| S1 | 26 | 32.70305 | 130.78224 | 48.4 |  |  |  | Ground surface | |  |
| S1 | 27 | 32.70503 | 130.78426 | 34.1 |  |  |  | Field | |  |
| S1 | 28 | 32.70825 | 130.78361 | 26.9 |  |  |  | Road | |  |
| S1 | 29 | 32.71264 | 130.78585 | 49.3 |  | R | 0.10-0.15 | Road | |  |
| S1 | 30 | 32.71445 | 130.80302 | 20.8 |  |  |  | Field | |  |
| S1 | 31 | 32.71420 | 130.80325 | 20.4 | 0.05-0.10 | L | 0.01-0.03 | Road | |  |
| S1 | 32 | 32.72208 | 130.81124 | 39.3 | 0.20 |  |  |  | |  |
| S1 | 33 | 32.72488 | 130.81156 | 43.2 | 0.10 |  |  | Road | |  |
| S1 | 34 | 32.72956 | 130.80009 | 44.8 | 0.10 |  |  | Road | |  |
| S1 | 35 | 32.73123 | 130.79888 | 51.7 |  |  |  | Road, Mole track | |  |
| S1 | 36 | 32.73512 | 130.79511 | 47.1 | 0.25 |  |  | Ground surface | |  |
| S1 | 37 | 32.73895 | 130.79605 | 31.7 |  | R | 0.60-0.62 | Field | |  |
| S1 | 38 | 32.73748 | 130.79560 | 35.2 |  | R | 0.25 | Field | | Site 4, Fig. 5(d) |
| S1 | 39 | 32.74884 | 130.79885 | 27.8 |  | R | 0.20-0.31 | Road | |  |
| S1 | 40 | 32.74527 | 130.79784 |  |  | R | 0.28 | Water way | |  |
| S1 | 41 | 32.75076 | 130.79944 |  |  | R | 0.29 | Field | |  |
| S1 | 42 | 32.75287 | 130.80052 |  |  |  |  |  | |  |
| S1 | 43 | 32.75315 | 130.80027 | 8.2 |  | R | 0.25 | Road | | Site 3, Fig. 5(c) |
| S1 | 44 | 32.75350 | 130.80105 | 5.3 | 0.05-0.10 | R |  | Field | |  |
| S1 | 138 | 32.72440 | 130.78691 |  |  | R | 0.02-0.03 | Road | |  |
| S1 | 139 | 32.72628 | 130.78589 | 7.9 |  |  |  | Field, Liquefaction | |  |
| S1 | 141 | 32.72119 | 130.78974 | 13.1 |  | R | 0.22 | Field | | Site 1, Fig. 5(a) |
| S1 | 142 | 32.73918 | 130.79720 | 33.5 |  |  |  | Field | | Site 2, Figs. 5(b), 6(a-b) |
| S2 | 1 | 32.76316 | 130.75298 |  | 0.20-0.30 |  |  | Road | |  |
| S2 | 2 | 32.76081 | 130.75414 | 6.4 |  |  |  | Liquefaction | |  |
| S2 | 3 | 32.76122 | 130.75464 | 6.8 |  | R | 0.15 | Field | |  |
| S2 | 4 | 32.75534 | 130.75397 | 3.5 | 0.15 |  |  | Bridge | |  |
| S2 | 5 | 32.75448 | 130.75188 | 1.6 | 0.15 |  |  | Bridge | |  |
| S2 | 6 | 32.75252 | 130.74674 | 1.3 |  |  |  | Field, Liquefaction | |  |
| S2 | 7 | 32.75245 | 130.74247 | 5.0 |  |  |  | Ground surface | | Site 8, Fig. 5(h) |
| S2 | 8 | 32.75196 | 130.74314 | 2.7 |  |  |  |  | | Site 6, Fig. 5(f) |
| S2 | 9 | 32.75623 | 130.77697 | 5.8 |  |  |  | Field | | Site 7, Fig. 5(g) |
| S2 | 10 | 32.76041 | 130.77459 | 4.6 | 0.10 |  |  | Field, Liquefaction | |  |
| S2 | 11 | 32.76367 | 130.77326 | 4.0 |  |  |  | Road | |  |
| S2 | 12 | 32.76341 | 130.78033 | 5.5 |  |  |  | Road | | Site 5, Fig. 5(e) |
| S2 | 13 | 32.76286 | 130.78890 | 6.6 |  |  |  | Road | |  |
| S2 | 14 | 32.75982 | 130.78913 | 9.7 |  |  |  | Ground surface | |  |
| S2 | 15 | 32.75636 | 130.78678 | 8.6 |  |  |  |  | |  |
| S2 | 16 | 32.75548 | 130.78679 | 8.9 |  | L | 0.10 | Road | |  |
| S2 | 18 | 32.75805 | 130.81163 | 6.4 |  |  |  | Road | |  |
| S | 17 | 32.74837 | 130.78723 | 32.4 | 0.40 |  |  | Road | |  |
| S | 69 | 32.73241 | 130.84881 | 262.4 |  |  |  | Road, Mole track | |  |
| S | 70 | 32.73069 | 130.84975 | 265.4 |  |  |  | Field | |  |
| S | 71 | 32.73021 | 130.84839 | 246.9 |  |  |  | Field | |  |
| S | 72 | 32.73055 | 130.84136 | 252.2 |  |  |  | Road | |  |
| S | 140 | 32.73063 | 130.78010 | 9.9 |  |  |  | Road | |  |
| SC | 22 | 32.76794 | 130.81744 | 3.4 |  |  |  | | Road, Mole track |  |
| SC | 23 | 32.76898 | 130.81838 | 3.3 |  | R | 1.30 | | Road |  |
| SC | 24 | 32.80179 | 130.85396 | 42.0 | 0.20-0.30 | R | 1.30 | | Road | Site 10, Fig. 7(a) |
| SC | 25 | 32.80194 | 130.85428 | 38.6 |  | R | 1.10 | | Ground surface | Site 11, Fig. 7(b) |
| SC | 45 | 32.76927 | 130.81805 | 3.8 |  |  |  | | Road |  |
| SC | 46 | 32.77492 | 130.81474 | 8.7 |  |  |  | | Bridge |  |
| SC | 47 | 32.78070 | 130.83251 | 20.9 | 0.15-.0.2 | R | 0.60-0.75 | | Road | Site 12, Fig. 7(c) |
| SC | 48 | 32.78089 | 130.83307 | 21.7 |  |  |  | | Field |  |
| SC | 49 | 32.78110 | 130.83382 | 25.0 |  | R | 0.62-0.75 | | Field |  |
| SC | 50 | 32.78126 | 130.83431 | 25.6 |  | R | 0.60 | | Mole track |  |
| SC | 51 | 32.78174 | 130.83619 | 32.9 | 0.30 | R | 0.80 | | Water way |  |
| SC | 52 | 32.78220 | 130.83491 | 25.8 |  |  |  | | Road |  |
| SC | 53 | 32.78214 | 130.83517 | 25.4 | 0.30-0.35 |  |  | | Road |  |
| SC | 54 | 32.78311 | 130.83674 | 46.0 |  | L | 0.30 | | Road |  |
| SC | 55 | 32.78520 | 130.84930 | 115.0 |  |  |  | | Field |  |
| SC | 56 | 32.78480 | 130.84988 | 120.3 |  |  |  | | Road |  |
| SC | 57 | 32.79555 | 130.84922 | 35.6 | 0.28 | R | 0.35 | | Road, Mole track | Site 16, Fig. 7(g) |
| SC | 58 | 32.79640 | 130.85097 | 37.0 | 0.26 | R | 0.46 | | Road |  |
| SC | 59 | 32.79667 | 130.85153 | 37.6 |  | R | 0.85 | | Field |  |
| SC | 60 | 32.79692 | 130.85247 | 42.9 | 0.28 |  | 0.65 | | Field | Site 12, Fig. 7(c) |
| SC | 61 | 32.79722 | 130.85298 | 44.8 | 0.30 | R | 0.60 | | Field |  |
| SC | 62 | 32.79738 | 130.85337 | 44.0 | 0.30 | R | 1.05 | | Field |  |
| SC | 63 | 32.79767 | 130.85315 | 41.1 |  | L | 0.26 | | Field |  |
| SC | 64 | 32.79860 | 130.85079 | 34.0 |  | L | 0.40 | | Field |  |
| SC | 65 | 32.80102 | 130.85181 | 32.9 |  | R |  | | Road |  |
| SC | 66 | 32.80086 | 130.85104 | 32.3 |  | R | 0.80 | | Field |  |
| SC | 67 | 32.80998 | 130.86670 | 86.9 |  | R | 0.65 | | Road |  |
| SC | 68 | 32.80919 | 130.86559 | 78.2 |  |  |  | | Mole track |  |
| SC | 68 | 32.73225 | 130.84933 | 261.7 |  |  |  | |  |  |
| SC | 68 | 32.73227 | 130.84932 | 261.7 |  |  |  | |  |  |
| SC | 68 | 32.73241 | 130.84920 | 261.0 |  |  |  | |  |  |
| SC | 73 | 32.77444 | 130.83749 | 87.1 |  | R L | 0.05 0.15 | | Road |  |
| SC | 74 | 32.82569 | 130.89188 | 138.6 | 0.10-0.15 | R | 0.05 | | Field, Graben |  |
| SC | 75 | 32.82288 | 130.89334 | 142.2 |  |  |  | | Field |  |
| SC | 76 | 32.82229 | 130.89255 | 147.2 |  |  |  | | Road |  |
| SC | 77 | 32.82374 | 130.89864 | 183.6 | 0.30 | L | 0.07, 0.40 | | Road |  |
| SC | 101 | 32.80498 | 130.85653 | 66.2 |  |  |  | | Outcrop |  |
| SC | 102 | 32.80612 | 130.85691 | 41.9 |  |  |  | | Ground surface |  |
| SC | 103 | 32.80471 | 130.85615 | 57.7 |  |  |  | | Outcrop |  |
| SC | 104 | 32.81145 | 130.86626 | 67.6 |  |  |  | | Road |  |
| SC | 105 | 32.81053 | 130.86658 | 83.3 |  | R | 0.10 | | Road |  |
| SC | 106 | 32.82222 | 130.89652 | 186.0 |  | L | 0.10 | | Road |  |
| SC | 107 | 32.82078 | 130.89641 | 203.7 | 0.08 | R | 0.10-0.11 | | Road |  |
| SC | 143 | 32.79416 | 130.84749 | 40.6 | 0.51 |  |  | | Field | Site 13, Fig. 7(d) |
| SC | 144 | 32.79504 | 130.84850 | 38.7 | 0.45 |  |  | | Ground surface |  |
| SC | 145 | 32.79552 | 130.84917 | 39.8 | 0.58 |  |  | | Road, Mole track |  |
| SC | 146 | 32.79658 | 130.85117 | 41.1 |  |  | 0.75 | | Field |  |
| SC | 147 | 32.79667 | 130.85156 | 42.9 |  | R | 0.75 | | Field |  |
| SC | 148 | 32.79697 | 130.85251 | 48.1 | 0.55 |  |  | | Field |  |
| SC | 149 | 32.79773 | 130.85307 | 48.2 | 0.24 | L | 0.69 | | Field |  |
| SC | 150 | 32.79736 | 130.85329 | 48.3 | 0.53 | R | 1.52 | | Water way |  |
| SC | 151 | 32.80529 | 130.85079 | 54.0 |  |  |  | | Outcrop |  |
| SC | 213 | 32.80429 | 130.85902 | 60.9 |  | R | 2.10-2.50 | | Field | Site 9, Figs. 6(c, d),  8(a-c) |
| SC | 214 | 32.80429 | 130.85902 | 65.5 |  |  |  | | Field |  |
| SC | 215 | 32.79896 | 130.84860 | 46.8 |  |  |  | | Field |  |
| SC | 222 | 32.81069 | 130.86334 | 51.1 |  |  |  | | Landslide |  |
| SC | 223 | 32.80802 | 130.86377 | 90.2 |  | R | 1.60 | | Path |  |
| SC | 224 | 32.78067 | 130.83254 | 51.2 |  | R | 0.55 | | Road |  |
| SC | 225 | 32.78080 | 130.83287 | 43.1 |  | R | 0.68 | | Field |  |
| SC | 226 | 32.78089 | 130.83320 | 42.9 | 0.35 | R | 0.72 | | Field |  |
| SC | 227 | 32.78098 | 130.83346 | 43.3 |  | R | 0.55 | | Field |  |
| SC | 228 | 32.78108 | 130.83370 | 43.0 |  | R | 0.58 | | Field |  |
| SC | 229 | 32.78110 | 130.83379 | 42.0 |  | R | 0.54 | | Field |  |
| SC | 230 | 32.78116 | 130.83393 | 42.3 |  | R | 0.75 | | Field |  |
| SC | 231 | 32.78121 | 130.83403 | 43.3 |  | R | 0.45 | | Field |  |
| SC | 232 | 32.78130 | 130.83456 | 42.0 |  | R | 0.65 | | Water way |  |
| SC | 233 | 32.78214 | 130.83516 | 38.4 |  | L | 0.42 | | Water way |  |
| SC | 234 | 32.78222 | 130.83490 | 36.9 | 0.40 | L | 0.42 | | Field | Site 15, Fig. 7(f) |
| SC | 235 | 32.78269 | 130.83352 | 32.0 |  | L | 0.55 | | Field | Site 14, Fig. 7(e) |
| SC | 236 | 32.78268 | 130.83386 | 32.7 | 0.20 | L | 0.63 | | Field |  |
| SC | 237 | 32.78029 | 130.82866 | 21.5 |  | R | 0.10 | | Road |  |
| SC | 238 | 32.78012 | 130.82994 | 23.3 |  | R | 0.80 | | Field |  |
| SC | 239 | 32.48111 | 130.51281 |  |  |  |  | | Outcrop |  |
| NC1 | 207 | 32.87680 | 130.94180 | 227.7 |  |  |  | | Road, Mole track |  |
| NC1 | 208 | 32.87691 | 130.95341 | 237.5 |  | R | 0.05-0.10 | | Road |  |
| NC1 | 209 | 32.87778 | 130.95741 | 255.5 | 0.40 | R | 0.70 | | Ground surface |  |
| NC1 | 211 | 32.87663 | 130.95981 |  |  | R | 0.30 | | Ground surface |  |
| NC1 | 212 | 32.87679 | 130.95506 | 251.3 |  | R | 0.05 | | Road |  |
| NC2 | 78 | 32.82739 | 130.90290 | 171.6 | 0.05-0.15 | L | 0.20 | | Field |  |
| NC2 | 79 | 32.83561 | 130.90983 | 180.9 |  |  |  | | Field |  |
| NC2 | 80 | 32.83541 | 130.91082 | 183.9 |  | L | 0.25 | | Ground surface |  |
| NC2 | 81 | 32.83584 | 130.91214 | 188.6 |  | R L | 0.05-0.35 0.05-0.30 | | Ground surface |  |
| NC2 | 82 | 32.83440 | 130.91494 | 232.1 |  | R | 1.33 | | Road |  |
| NC2 | 83 | 32.83405 | 130.91464 | 227.2 | 0.25 | R | 0.56 | | Water way |  |
| NC2 | 84 | 32.83399 | 130.91449 | 226.8 |  | R | 1.26 | | Field |  |
| NC2 | 85 | 32.84255 | 130.92638 | 220.7 |  |  |  | | Ground surface |  |
| NC2 | 85 | 32.84262 | 130.92707 | 222.1 |  | L | 1.00 | | Bridge |  |
| NC2 | 87 | 32.84140 | 130.93182 | 249.4 |  | R | 0.90 | | Ground surface |  |
| NC2 | 153 | 32.83406 | 130.91471 | 242.3 |  |  |  | |  |  |
| NC2 | 154 | 32.83359 | 130.91356 | 249.0 |  | R | 1.30 | | Field |  |
| NC2 | 194 | 32.84178 | 130.91566 | 187.2 |  |  |  | | Road |  |
| NC3 | 86 | 32.83108 | 130.94211 | 410.8 |  | R | 2.45 | | Ground surface | Sites 18-19, Fig. 8(d-g) |
| NC3 | 108 | 32.82319 | 130.90457 | 283.4 | 0.40 | R | 0.40 | | Field |  |
| NC3 | 109 | 32.82327 | 130.90724 | 282.7 | 0.13 | R | 0.10 | | Field |  |
| NC3 | 152 | 32.50039 | 130.54538 | 232.1 | 0.25 | R | 1.30 | | Road | Site 17, Fig. 7(c) |
| NC3 | 188 | 32.81922 | 130.91588 | 320.9 |  |  |  | | Road, Landslide |  |
| NC3 | 189 | 32.82423 | 130.91414 | 277.8 |  |  |  | | Landslide |  |
| NC3 | 190 | 32.82064 | 130.92148 | 310.5 | 0.60 | R | 0.50 | | Road |  |
| NC3 | 191 | 32.82227 | 130.92685 | 324.2 | 1.10 | R | 0.80 | Road | |  |
| NC3 | 192 | 32.82432 | 130.92897 | 312.5 |  |  |  | Ground surface | |  |
| NC3 | 193 | 32.82441 | 130.92996 | 326.4 |  | R | 0.20 | Road | |  |
| NC3 | 783 | 32.82727 | 130.91537 | 257.2 |  |  |  |  | |  |
| N | 97 | 32.88826 | 131.01162 | 537.0 |  | R | 0.30 | Road | |  |
| N1 | 170 | 32.92488 | 130.99596 | 488.6 | 0.50 | L | 0.60 | Road | |  |
| N1 | 171 | 32.92434 | 130.99631 | 484.6 | 0.40 | L | 0.50 | Field, Road | |  |
| N1 | 172 | 32.95644 | 131.03680 | 485.0 | 0.80-1.45 | L | 0.16, 0.20 | Road, Graben | |  |
| N1 | 173 | 32.95608 | 131.03641 | 483.4 | 1.25-1.35 | L | 0.40, 0.50 | Field | | Site 21, Fig. 9(c) |
| N1 | 174 | 32.95613 | 131.03572 | 481.6 | 0.80 | R | 0.50 | Field | | Site 22, Fig. 9(d) |
| N1 | 174 | 32.94290 | 131.08016 | 517.6 |  |  |  | Road, small mole track | |  |
| N1 | 177 | 32.92877 | 131.01307 | 489.9 |  |  |  | Road | |  |
| N1 | 179 | 32.91592 | 130.98137 | 473.3 |  | L | 0.18 | Road | |  |
| N1 | 180 | 32.91606 | 130.98182 | 473.4 | 0.20 |  |  | Road | |  |
| N1 | 181 | 32.91602 | 130.97850 | 470.3 | 0.10 |  |  | Field, Liquefaction | |  |
| N1 | 182 | 32.91680 | 130.97013 | 610.0 |  |  |  | Road | |  |
| N1 | 183 | 32.91713 | 130.97862 | 475.4 |  |  |  | Road | |  |
| N1 | 184 | 32.91595 | 130.98664 | 474.1 | 0.22 | L | 0.18 | Road | |  |
| N1 | 185 | 32.91786 | 130.98748 | 473.3 |  |  |  | Road | |  |
| N1 | 186 | 32.91749 | 130.98859 | 471.5 | 0.20-0.25 |  |  | Road | |  |
| N1 | 195 | 32.94329 | 131.01985 | 480.0 | 0.25-0.30 | L | 0.65 | Field | |  |
| N1 | 196 | 32.94494 | 131.01698 | 472.3 | 0.80 |  |  | Field, Graben | |  |
| N1 | 196 | 32.94481 | 131.01633 | 471.6 |  |  |  | Field, Graben | |  |
| N1 | 196 | 32.94558 | 131.01749 | 472.0 | 0.60 |  |  | Field, Graben | |  |
| N1 | 197 | 32.97279 | 131.05006 | 478.5 |  |  |  | Ground surface | |  |
| N1 | 198 | 32.97507 | 131.05206 | 481.8 |  |  |  | Ground surface | |  |
| N1 | 199 | 32.98104 | 131.05148 | 478.5 |  |  |  | Road | |  |
| N1 | 200 | 32.97821 | 131.05079 | 479.8 | 0.85-0.90 |  |  | Field | |  |
| N1 | 201 | 32.97631 | 131.04911 | 482.1 | 0.40-0.43 |  |  | Field | |  |
| N1 | 204 | 32.94494 | 131.02854 | 482.9 | 0.15-0.20 |  |  | Road | |  |
| N1 | 205 | 32.94877 | 131.01849 | 471.6 | 0.30-0.40 | R | 0.70 | Water way | |  |
| N1 | 206 | 32.92833 | 131.00216 | 474.7 | 0.25 | L | 0.40 | road | |  |
| N1 | 240 | 32.92909 | 131.00214 |  |  |  |  | Field | |  |
| N1 | 241 | 32.92818 | 131.00011 |  | 1.75 |  |  | Field | | Site 20, Fig. 9(a-b) |
| N1 | 242 | 32.95269 | 131.02309 |  |  |  |  | Field | |  |
| N2 | 117 | 32.90833 | 130.99361 | 475.3 |  | R | 0.10 | Road | | Site 24, Fig. 9(f) |
| N2 | 178 | 32.90131 | 130.98958 | 478.9 |  |  |  | Road | | Site 23, Fig. 9(e) |
| N2 | 187 | 32.91183 | 131.00278 | 515.8 |  | L | 0.05-0.10 | Road | |  |
| N3 | 98 | 32.89493 | 31.00505 | 533.7 |  | R | 1.80 | Road | |  |
| N3 | 99 | 32.89412 | 131.00271 | 520.6 |  | R | 0.10 | Road, Mole track | |  |
| N3 | 116 | 32.89474 | 131.00520 | 543.8 |  | R | 0.15 | Road | |  |
| N3 | 118 | 32.90696 | 131.02494 | 660.7 |  | R | 0.49 | Ground surface | |  |
| N3 | 119 | 32.90387 | 131.04298 | 818.5 | 0.90 | L | 0.95 | Ground surface | |  |
| N3 | 120 | 32.90478 | 131.04205 | 832.5 |  | L | 0.58 | Ground surface | | Site 28, Fig. 10(e) |
| N3 | 121 | 32.90631 | 131.04198 | 881.8 |  |  |  |  | | Site 30, Fig. 10(h) |
| N3 | 122 | 32.90531 | 131.04406 |  |  |  |  | Ground surface | | Site 27, Fig. 10 (b, d, f) |
| N3 | 123 | 32.90908 | 131.04363 | 840.4 |  |  |  | Ground surface | |  |
| N3 | 131 | 32.91150 | 131.05363 | 812.9 |  | R | 0.15 | Ground surface | |  |
| N3 | 132 | 32.91132 | 131.05139 | 807.3 |  |  |  | Path, Mole track | |  |
| N3 | 133 | 32.91027 | 131.04773 | 833.9 |  |  |  |  | |  |
| N3 | 134 | 32.90560 | 131.04633 | 867.9 | 0.55 |  |  | Ground surface | |  |
| N3 | 135 | 32.90318 | 131.04536 | 869.7 |  |  |  | Ground surface | |  |
| N3 | 136 | 32.90321 | 131.03007 | 722.1 |  | L | 0.05 | Road | |  |
| N3 | 137 | 32.90006 | 131.02985 | 703.1 |  |  |  | Road | |  |
| N3 | 165 | 32.89306 | 131.00106 | 513.8 | 0.20 | R | 0.70 | Road | |  |
| N3 | 166 | 32.89031 | 130.99738 | 482.7 |  | L | 0.37 | Field | | Site 25, Fig. 9(g) |
| N3 | 167 | 32.89016 | 130.99670 | 482.4 | 0.37 | L | 0.05-0.20 | Field | |  |
| N3 | 168 | 32.89063 | 130.99540 | 485.1 |  | R | 0.15 | Road | |  |
| N3 | 169 | 32.89143 | 130.99591 | 489.0 |  | R | 0.70 | Ground surface, Mole track | | Site 26, Fig. 9(h) |
| N3 | 175 | 32.94315 | 131.07997 | 519.2 |  |  |  | Field | |  |
| N3 | 176 | 32.94343 | 131.08015 | 515.6 |  | R | 0.05 | Field | | Site 29, Fig. 10(g) |
| N3 | 202 | 32.96201 | 131.08075 | 491.0 | 0.30 |  |  | Road, Field | | Site 31, Fig. 11(a-b) |
| N3 | 210 | 32.87874 | 130.97374 | 321.6 |  |  |  | Ground surface, Landslide | |  |
| N4 | 88 | 32.87683 | 130.99043 | 426.0 |  |  |  | Ground surface | |  |
| N4 | 89 | 32.87637 | 131.00502 | 487.6 |  |  |  | Road | |  |
| N4 | 90 | 32.87871 | 131.00897 | 517.3 | 0.29 | R | 0.52 | Road | |  |
| N4 | 91 | 32.87822 | 131.00417 | 500.0 | 0.30 | R | 0.30 | Road, Field | |  |
| N4 | 92 | 32.87982 | 131.00350 | 496.6 | 0.39 | R L | 0.20 0.20 | Road | |  |
| N4 | 93 | 32.87935 | 131.00177 | 500.9 |  |  |  | Ground surface | |  |
| N4 | 93 | 32.87904 | 131.00122 | 501.1 |  |  |  |  | |  |
| N4 | 94 | 32.87719 | 130.98887 | 414.7 |  | R | 0.30 | Road | | Site 32, Fig. 11(c) |
| N4 | 95 | 32.88089 | 131.01363 | 534.8 |  | R | 0.05 | Road | |  |
| N4 | 96 | 32.88391 | 131.01451 | 545.5 |  |  |  | Ground surface | |  |
| N4 | 113 | 32.87612 | 131.00496 | 481.4 |  | R L | 0.30 0.47 | Ground surface | |  |
| N4 | 114 | 32.89189 | 131.02752 | 666.1 |  | R L | 0.05 0.07 | Road | |  |
| N4 | 115 | 32.89392 | 131.02710 | 673.1 | 0.30-0.40 |  |  | Road | |  |
| N4 | 124 | 32.89876 | 131.04611 | 937.8 |  |  |  | Landslide | |  |
| N4 | 125 | 32.89838 | 131.04651 | 941.4 |  | L | 0.05 | Road | |  |
| N4 | 126 | 32.90002 | 131.05166 | 950.2 |  |  |  |  | |  |
| N4 | 126b | 32.90002 | 131.05166 |  |  |  |  |  | |  |
| N4 | 127 | 32.90309 | 131.05286 | 941.6 |  |  |  | Landslide | |  |
| N4 | 128 | 32.90434 | 131.05476 | 934.5 | 0.40 | R | 0.50 | Road | |  |
| N4 | 129 | 32.90863 | 131.06104 | 895.4 |  | R | 0.05 | Road | |  |
| N4 | 130 | 32.91135 | 131.06485 | 875.6 |  |  |  | Landslide | |  |
| N4 | 159 | 32.88140 | 131.01236 | 522.6 |  |  |  | Field | | Site 34, Fig. 11(e-h) |
| N4 | 160 | 32.88623 | 131.01706 | 566.1 | 0.39 |  |  | Field | |  |
| N4 | 161 | 32.88553 | 131.01659 | 578.4 | 0.59 | R | 0.55 | Field | |  |
| N4 | 162 | 32.88507 | 131.01631 | 577.3 | 0.10-0.20 |  |  | Field | |  |
| N4 | 163 | 32.88488 | 131.01561 | 572.1 | 0.25 | R | 0.35 | Road, Landslide | |  |
| N4 | 164 | 32.88840 | 131.02158 | 616.6 |  |  |  | Road | |  |
| N4 | 203 | 32.94818 | 131.11668 | 511.3 |  |  |  | Ground surface | |  |
| N5 | 110 | 32.87051 | 130.99455 | 385.5 |  | R | 0.02, 0.08 | Road | |  |
| N5 | 111 | 32.87142 | 130.99181 | 345.2 |  |  |  |  | |  |
| N5 | 112 | 32.86828 | 130.99180 | 319.1 |  |  |  | Landslide | |  |
| N5 | 155 | 32.85602 | 131.01533 | 395.9 |  |  |  | Road | |  |
| N5 | 156 | 32.86070 | 131.00294 | 371.8 |  |  |  | Ground surface | |  |
| N5 | 157 | 32.86386 | 130.99986 | 366.6 |  |  |  | | Outcrop |  |
| N5 | 158 | 32.87031 | 130.99568 | 405.1 |  |  |  | | Landslide | Site 35, Fig. 12(a,b) |
| N5 | 216 | 32.85464 | 131.01901 | 413.8 |  |  |  | | Outcrop | Site 36, Fig. 12(c,d) |
| N5 | 217 | 32.84641 | 131.03597 | 456.8 |  |  |  | | Road |  |
| N5 | 218 | 32.84450 | 131.04275 | 474.4 |  |  |  | | Field | Site 37, Fig. 12(e-g) |
| N5 | 218 | 32.84488 | 131.04315 | 476.8 |  |  |  | | Field |  |
| N5 | 219 | 32.84350 | 131.04666 | 470.0 |  |  |  | | Field |  |
| N5 | 220 | 32.84235 | 131.05457 | 517.3 |  |  |  | | Road | Site 39, Fig 12(h) |
| N5 | 221 | 32.84098 | 131.08162 | 591.5 |  |  |  | | Fault scarp |  |
| Seg., segment; S1, Southwest segment-Zone 1; S2, Southwest segment-Zone 2; SC, SW-central segment;  NC, NE-central segment; NC1, NE-central segment-Zone 1; NC2, NE-central segment-Zone 2;  NC3, NE-central segment-Zone 3; N1, Northeast-Zone 1; N2, Northeast-Zone 2; N3, Northeast-Zone 3;  N4, Northeast-Zone 4; N5, Northeast-Zone 5; No., location number; V, vertical offset; H, horizontal offset;  L, left-lateral component; R, right-lateral component;  Sites 1–35, main locations where the structural features of the co-seismic surface ruptures are shown in Figs. 5–12. | | | | | | | | | | |
|  |  |  |  |  |  |  |  |  |  |  |
|  |  |  |  |  |  |  |  |  |  |  |
|  |  |  |  |  |  |  |  |  |  |  |
